# A Downy Mildew Effector Attenuates Salicylic Acid–Triggered Immunity in Arabidopsis by Interacting with the Host Mediator Complex

**DOI:** 10.1371/journal.pbio.1001732

**Published:** 2013-12-10

**Authors:** Marie-Cécile Caillaud, Shuta Asai, Ghanasyam Rallapalli, Sophie Piquerez, Georgina Fabro, Jonathan D. G. Jones

**Affiliations:** 1 The Sainsbury Laboratory, John Innes Centre, Norwich, United Kingdom; 2 Center for Sustainable Resource Science, RIKEN, Yokohama, Kanagawa, Japan; Michigan State University, United States of America

## Abstract

Plants are continually exposed to pathogen attack but usually remain healthy because they can activate defences upon perception of microbes. However, pathogens have evolved to overcome plant immunity by delivering effectors into the plant cell to attenuate defence, resulting in disease. Recent studies suggest that some effectors may manipulate host transcription, but the specific mechanisms by which such effectors promote susceptibility remain unclear. We study the oomycete downy mildew pathogen of Arabidopsis, *Hyaloperonospora arabidopsidis* (*Hpa*), and show here that the nuclear-localized effector HaRxL44 interacts with Mediator subunit 19a (MED19a), resulting in the degradation of MED19a in a proteasome-dependent manner. The Mediator complex of ∼25 proteins is broadly conserved in eukaryotes and mediates the interaction between transcriptional regulators and RNA polymerase II. We found MED19a to be a positive regulator of immunity against *Hpa*. Expression profiling experiments reveal transcriptional changes resembling jasmonic acid/ethylene (JA/ET) signalling in the presence of HaRxL44, and also 3 d after infection with *Hpa*. Elevated JA/ET signalling is associated with a decrease in salicylic acid (SA)–triggered immunity (SATI) in *Arabidopsis* plants expressing HaRxL44 and in *med19a* loss-of-function mutants, whereas SATI is elevated in plants overexpressing *MED19a*. Using a *PR1*::GUS reporter, we discovered that *Hpa* suppresses *PR1* expression specifically in cells containing haustoria, into which RxLR effectors are delivered, but not in nonhaustoriated adjacent cells, which show high *PR1*::GUS expression levels. Thus, HaRxL44 interferes with Mediator function by degrading MED19, shifting the balance of defence transcription from SA-responsive defence to JA/ET-signalling, and enhancing susceptibility to biotrophs by attenuating SA-dependent gene expression.

## Abstract 2

HaRxL44, a secreted effector from the Arabidopsis downy mildew pathogen *Hyaloperonospora arabidopsidis*, enhances disease susceptibility by interacting with and degrading Mediator subunit MED19a, thereby perturbing plant defense gene transcription.

## Author Summary

The highly conserved Mediator complex plays an essential role in transcriptional regulation by providing a molecular bridge between transcription factors and RNA polymerase II. Recent studies in Arabidopsis have revealed that it also performs an essential role in plant defence. However, it remains unknown how pathogens manipulate Mediator function in order to increase a plant's susceptibility to infection. In this article, we show that a secreted effector, HaRxL44, from the Arabidopsis downy mildew pathogen *Hyaloperonospora arabidopsidis* (*Hpa*), interacts with and degrades the Mediator subunit MED19a, resulting in the alteration of plant defence gene transcription. This effector-mediated interference with host transcriptional regulation perturbs the balance between jasmonic acid/ethylene (JA/ET) and salicylic acid (SA)–dependent defence. HaRxL44 interaction with MED19a results in reduced SA-regulated gene expression, indicating that this pathogen effector modulates host transcription to promote virulence. The resulting alteration in defence transcription patterns compromises the plant's ability to defend itself against pathogens, such as *Hpa*, that establish long-term parasitic interactions with living host cells via haustoria (a pathogen structure that creates an expanded host/parasite interface to extract nutrients) but not against necrotrophic pathogens that kill host cells. HaRxL44 is unlikely to be the sole effector that accomplishes this shift in hormonal balance, and other nuclear HaRxL proteins were reported by other researchers to interact with Mediator components, as well as with other regulators of the JA/ET signalling pathway. Functional analyses of these effectors should facilitate the discovery of new components of the plant immune system. These data show that pathogens can target fundamental mechanisms of host regulation in order to tip the balance of signalling pathways to suppress defence and favour parasitism.

## Introduction

Plants and microbial pathogens co-evolve; pathogens are selected to evade host defence, and plants are selected to detect and resist pathogens [1],[2]. Resistance mechanisms include not only pattern-triggered immunity (PTI) and effector-triggered immunity (ETI) [1], but also local and systemic plant defence responses that are controlled through distinct, but partially interconnected pathways involving the hormones salicylic acid (SA) and jasmonic acid (JA)/ethylene (ET) [3]. Adapted pathogens have a substantial repertoire of effectors that can suppress PTI by various mechanisms [4] but only one effector has been shown to interfere with SA-triggered immunity (SATI) [5]. An important role in plant defence has been attributed to nuclear processes, since there are many reports that nuclear localisation of pathogen effectors, R proteins, and key host components, including transcription factors and regulators, is essential for plant immunity [6]. This observation suggests that effectors may manipulate host transcription or other nuclear regulatory components for the benefit of the pathogen.

Although filamentous phytopathogens such as fungal rusts and powdery mildews and oomycete downy mildews and white rusts are more damaging to agriculture than bacteria, their effector functions are more poorly understood. Fungal and oomycete effectors are secreted, and then taken up by the host cell via a poorly understood mechanism that for many oomycetes involves the N-terminal RxLR motif [7],[8]. Sequencing of several oomycete genomes including the model organism *Arabidopsis* downy mildew *Hyaloperonospora arabidopsidis* (*Hpa*) has allowed prediction of a repertoire of effector candidate genes that share N-terminal sequence motifs with known effectors [9],[10]. To establish an inventory of the *Hpa* RXLR effectors (HaRxLs), the draft genome of *Hpa* Emoy2 was scanned and HaRxL effector candidates were cloned. Because transformation of biotrophic pathogens such as *Hpa* is difficult, we developed heterologous systems to assess HaRxL functions [11],[12]. We first deployed a *Pseudomonas syringae* pv. tomato (*Pst*) type three secretion (T3S)–based delivery system (EDV) to look for HaRxLs that enhance *Pst* virulence and/or that suppress host defence outputs such as callose deposition, in order to prioritize effectors for follow-up studies [13],[14]. We next screened for the subcellular localisation of the HaRxL collection and identified 15 HaRxL effectors that localise to the plant cell nucleus when stably expressed in *Arabidopsis* [15],[16] and interact in yeast with nuclear plant proteins implicated in transcription [16],[17]. In particular, in yeast 2-hybrid (Y2H) assays, HaRxL44 interacts with MED19a, a subunit of the *Arabidopsis* Mediator complex [18]. Six other *Hpa* effectors interact with host Mediator or regulators of Mediator ([18]; Figure S1).

Mediator is a conserved multisubunit complex that acts as a molecular bridge between transcriptional regulators at gene enhancer sequences and the activation of transcription by RNA polymerase II at the transcription start site [18],[19]. Eight of 10 essential Mediator genes conserved between *S. pombe* and *S. cerevisiae* (including *MED19*) also have a metazoan homologue, indicating that a Mediator core has been conserved throughout evolution and is present in all eukaryotic cells [20]. Mediator is a large complex (>25 components), but different subunits are implicated in integration of specific external stimuli [21],[22]. Mediator has numerous functions in addition to interacting directly with RNA polymerase II as it can interact with and coordinate the action of many other co-activators and co-repressors, including those acting on chromatin [20]. These interactions ultimately allow the Mediator complex to deliver outputs ranging from the maximal activation of genes, through the modulation of basal transcription, to long-term epigenetic silencing [20]. Despite the importance of Mediator, this complex has been little studied, due to the lethality of mutants in most multicellular organisms. However, null mutations of Mediator subunit genes are often not lethal in plants, making these organisms a valuable model for studying the Mediator complex. In *Arabidopsis*, several Mediator subunits have been shown to have a specific function in the activation of signalling pathways during plant development and in response to abiotic stress. MED12/CRP (CRYPTIC PRECOCIOUS) and MED13/MAB2 (MACCHI-BOU2) are required for early embryo patterning, and also regulate flowering and cotyledon organogenesis, respectively [23],[24]. MED14/SWP (STRUWWELPETER) is a key regulator of cell proliferation [25]. MED16/SFR6 (SENSITIVE TO FREEZING6) integrates cellular and environmental cues into the circadian clock [26]–[28] and is required for cold acclimation. MED17, MED18, and MED20a play an important role in the production of small and long noncoding RNAs [29]. MED25/PFT1 (PHYTOCHROME AND FLOWERING TIME1) was first identified as a key regulator of flowering [30] and later found to regulate final organ size and light signalling [31],[32]. MED33a/RFR1 (REF4-RELATED1) and MED33b/REF4 (REDUCED EPIDERMAL FLUORESCENCE4) are required for phenylpropanoid homeostasis [33].

Mediator was recently shown to play a role in plant immunity and pest resistance. It was initially shown to be important for the activation of JA/ET-dependent defences against necrotrophic pathogens, via MED21 and MED25 [34],[35]. Other studies reveal a role for Mediator in the activation of SATI [36]. The Mediator subunits MED14, MED15, and MED16 have all been reported to be required for the biological induction of systemic acquired resistance (SAR) [37]–[39], suggesting that the Mediator may function in SAR activation. Both MED14 and MED15 appear to function downstream of NPR1 and do not affect the nuclear localisation or stability of NPR1 [37],[39], whereas MED16 makes a positive contribution to the accumulation of NPR1 protein [39]. The Mediator complex thus appears to be a “hub” for the plant immune system, but little is known about how the pathogen manipulates its function to promote disease.

We report here the functional analysis of a nuclear downy mildew effector, HaRxL44, which interacts with Mediator subunit 19a (MED19a), and causes its degradation via proteasome-mediated degradation of this subunit. Expression profiling revealed an induction of JA/ET signalling in the presence of HaRxL44, mimicking that observed after 3 d of compatible interaction. This increase in JA/ET signalling was associated with low levels of SATI in both *Arabidopsis* plants expressing HaRxL44 and in *med19a* knock-out mutants, whereas high levels of SATI were observed in plants overexpressing *MED19a*. Using the *PR1*::GUS reporter, we confirmed that *Hpa* abolishes *PR1* expression specifically in cells containing haustoria. Thus, HaRxL44 affects via MED19a the balance between JA/ET and SA signalling and thus enhances biotroph susceptibility.

## Results

### HaRxL44 Targets MED19a, a Positive Regulator of Plant Immunity to *Hpa*

**Figure 1:**
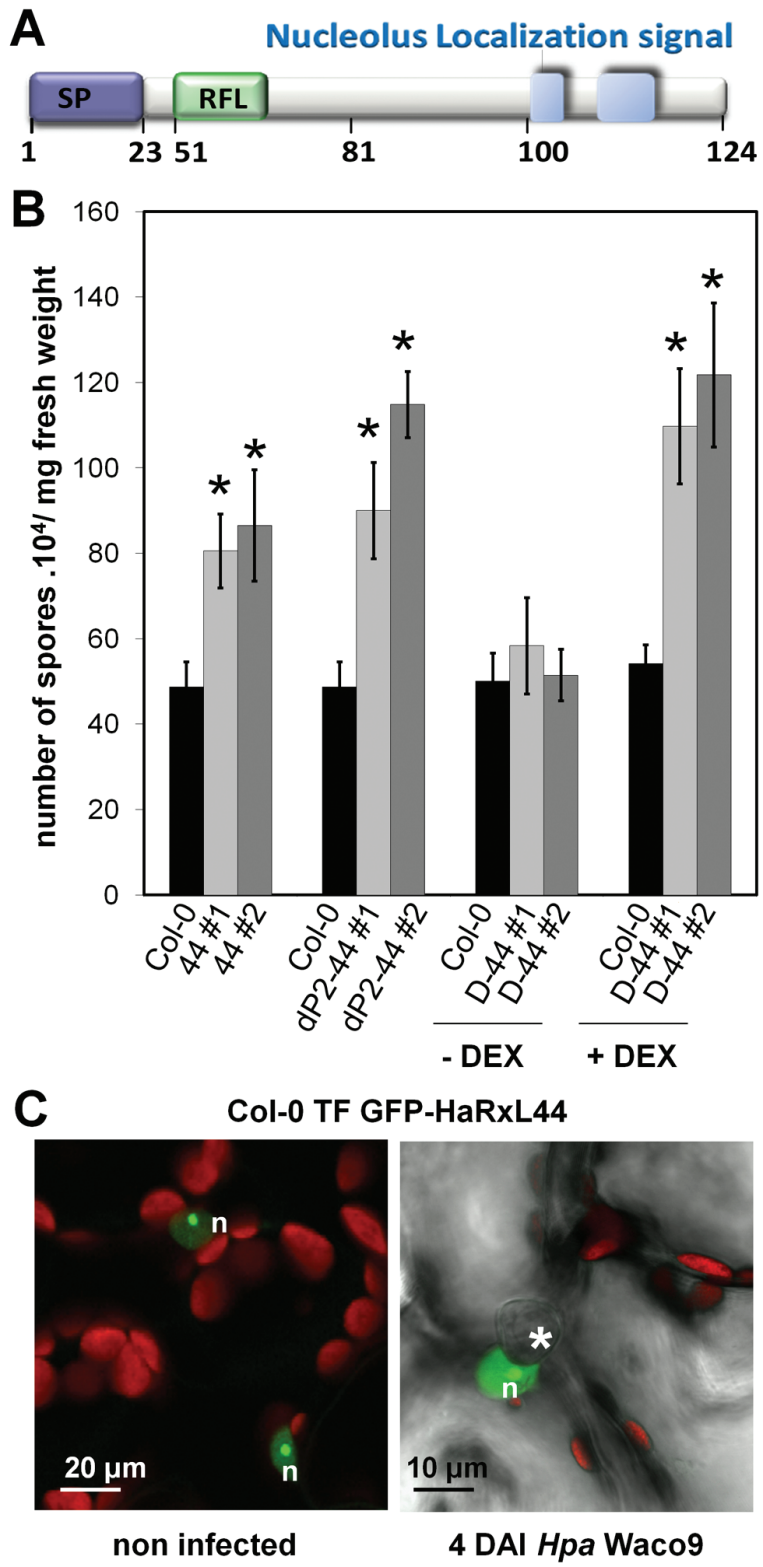
HaRxL44 is a nuclear-HaRxL that enhances plant susceptibility to *Hpa.* (A) *In silico* prediction of HaRxL44 protein organization. SP, signal peptide; RFL, RxLR motif. (B) Monitoring of *Hpa* Waco9 sporulation at 5 d after inoculation in transgenic lines expressing HaRxL44 under the control of 35S promoter (44 lines), under the control of an “haustoriated-cell specific” promoter (dP2–44 lines), under the control of DEX inducible promoter (D44 lines). For D44 lines, plants were treated with DEX 2d after *Hpa* infection, in order to induce HaRxL44 expression. Expression of HaRxL44 in all the lines was monitored by Western blot (see Figure S3). Error bars represent the standard error of the mean. Asterisks represent the significance of individual unpaired *t* tests comparing the given column with the control. (C) Subcellular localisation of GFP-HaRxL44 4 DAI with *Hpa*. The green colour corresponds to the GFP signal, and the red colour corresponds to chloroplast autofluorescence. Asterisks indicate the position of the haustorium. n, nucleus.

In a previous functional screen for *Hpa* virulence factors, we identified HaRxL44 (Figure 1A) as an enhancer of bacterial virulence in *Arabidopsis* [13]. The amino-acid sequence of HaRxL44 displays similarity to two predicted RXLR effectors from *Phytophthora infestans*, PITG-04266 and PITG_07586, and avh109 from *P. sojae* (Figure S2A). As observed for its homolog PITG_07586 from the “plastic secretome” of *P. infestans* [40], *HaRxL44* is found in a region of the *Hpa* genome enriched in retrotransposons (Figure S2B) and is conserved between *Hpa* races (Figure S2C). We confirmed the effect of HaRxL44 on virulence (Figure 1B) by generating transgenic lines of *Arabidopsis* expressing *HaRxL44* under the control of various promoters (Figure S3). Subcellular localization of GFP-HaRxL44 in a stably transformed Arabidopsis line (Figure 1C) confirmed its nuclear localization during *Hpa* infection, during which the nucleus is found closely associated with *Hpa* haustoria [15].

**Figure 2:**
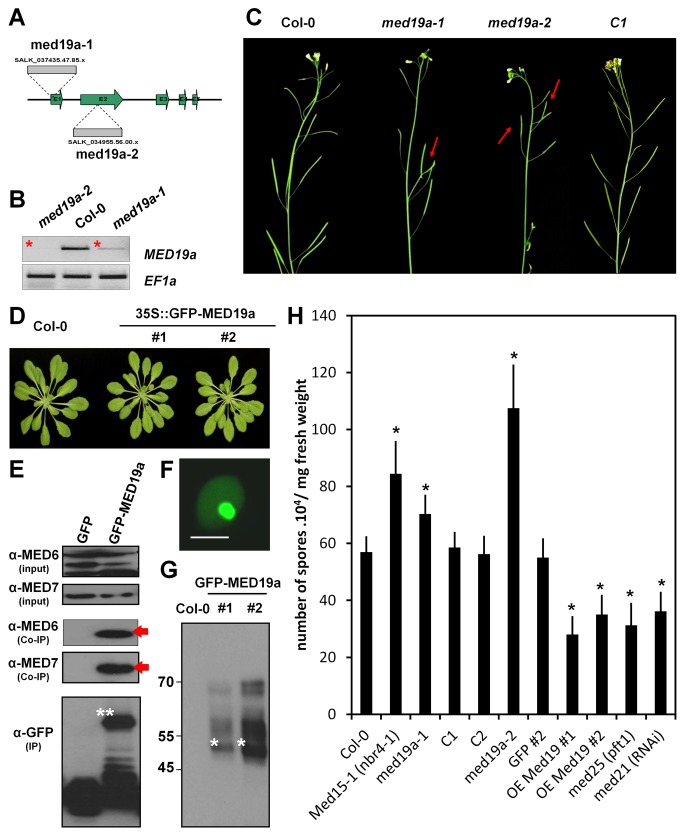
MED19a is a positive regulator of nuclear immunity against *Hpa*. (A) Schematic diagram of T-DNA insertions in *MED19a*. (B) *MED19a* expression in *med19a-1* and *med19a-2* mutants. (C) Representative images of the phenotype observed in 4-wk-old floral stem of Col-0, *med19a-1*, *med19a-2*, and *med19a* mutant complemented line C1. (D) Developmental phenotype of *Arabidopsis* transgenic lines OE-MED19a compared to Col-0. (E) Immunoblot of the Co-immunoprecipitation analysis between GFP-MED19a and MED6 and MED7. Arrows point out the interaction detected between GFP-MED19a and MED6 and MED7. (F) Subcellular localisation of GFP-MED19a in *Arabidopsis* plant. Scale bar, 5 µm. (G) Immunoblot of proteins extracted from two independent lines expressing GFP-MED19a. Stars indicate the expected size for GFP-MED19a. Notice the upper bands in the blot that might suggest posttranscriptional modifications. (H) Monitoring of *Hpa* sporulation at 5 DAI in control lines (Col-0 and GFP), *med19a* mutant complemented lines (C1 and C2), Mediator mutants, and MED19a OE lines. Error bars represent the standard error of the mean. Asterisks represent the significance of individual unpaired *t* tests comparing the given column with the control (*p* value<0.01).

In an extensive Y2H screen [17], HaRxL44 was found to interact with several nuclear proteins, including MED19a (Figure S3A, S3B). We assessed the functional role of Mediator in immunity to *Hpa*, by studying the contribution of MED19a during *Hpa* infection. We first isolated *med19a* loss-of-function alleles (Figure 2A, 2B) and found that *med19a* mutant plants had a wild-type (WT) phenotype, with the exception of abnormally shaped siliques (Figure 2C). In parallel, we generated Col-0 *Arabidopsis* transgenic lines overexpressing a construct encoding *MED19a* fused to a GFP tag (OE MED19a; Figure 2D). Homozygous *med19a-1* and *med19a-2* mutants expressing GFP-MED19a were produced in order to check for complementation. We tested by Western blot the expression of GFP-MED19a in the mutant background, and selected lines with lower expression levels than observed for OE MED19a lines (C1, C2; Figure S4). In these selected lines, GFP-MED19a rescued the phenotype observed during plant development (Figure 2C).

We confirmed that the fusion protein was functional, by checking that GFP-MED19a interacted with the Mediator complex. Immunoprecipitation of the GFP-MED19a protein in *Arabidopsis* led to the detection of both MED6 and MED7 in pull-down assays with native antibodies (Figure 2E).

We then analysed the subcellular localisation of MED19a *in vivo* in *Arabidopsis* by confocal microscopy. Live-cell imaging showed that GFP-MED19a and HaRxL44 were present in the same compartments: the nucleoplasm and nucleolus of the plant cell (Figure 2F). Western-blot analysis of two independent transgenic lines producing GFP-MED19a (Figure 2G) demonstrated the presence of a GFP-MED19a protein of the expected size (50 kDa), together with additional signals at higher molecular weights (60 kDa and 70 kDa), suggesting that MED19a is modified post-translationally *in planta*.

We then challenged the transgenic lines with *Hpa* and monitored pathogen growth after six days. Both the *med19a-1* and *med19a-2* mutants were more susceptible to *Hpa* than wild type, similar to a *med15* mutant, which has impaired SATI (*med15* [37]; Figure 2H). Complemented lines displayed the same level of susceptibility as wild type plants (Figure 2H), confirming the functionality of the fusion protein. By contrast, transgenic lines overproducing MED19a were more resistant to *Hpa* than the WT or *Arabidopsis* lines expressing GFP alone. Therefore, GFP-MED19a can associate with other Mediator subunits and complements *med19a* loss of function alleles, which suggests that the fusion protein is functional. Thus, the Mediator subunit MED19a is a positive regulator of plant immunity to *Hpa*.

### HaRxL44 destabilizes MED19a in a proteasome-dependent manner

**Figure 3:**
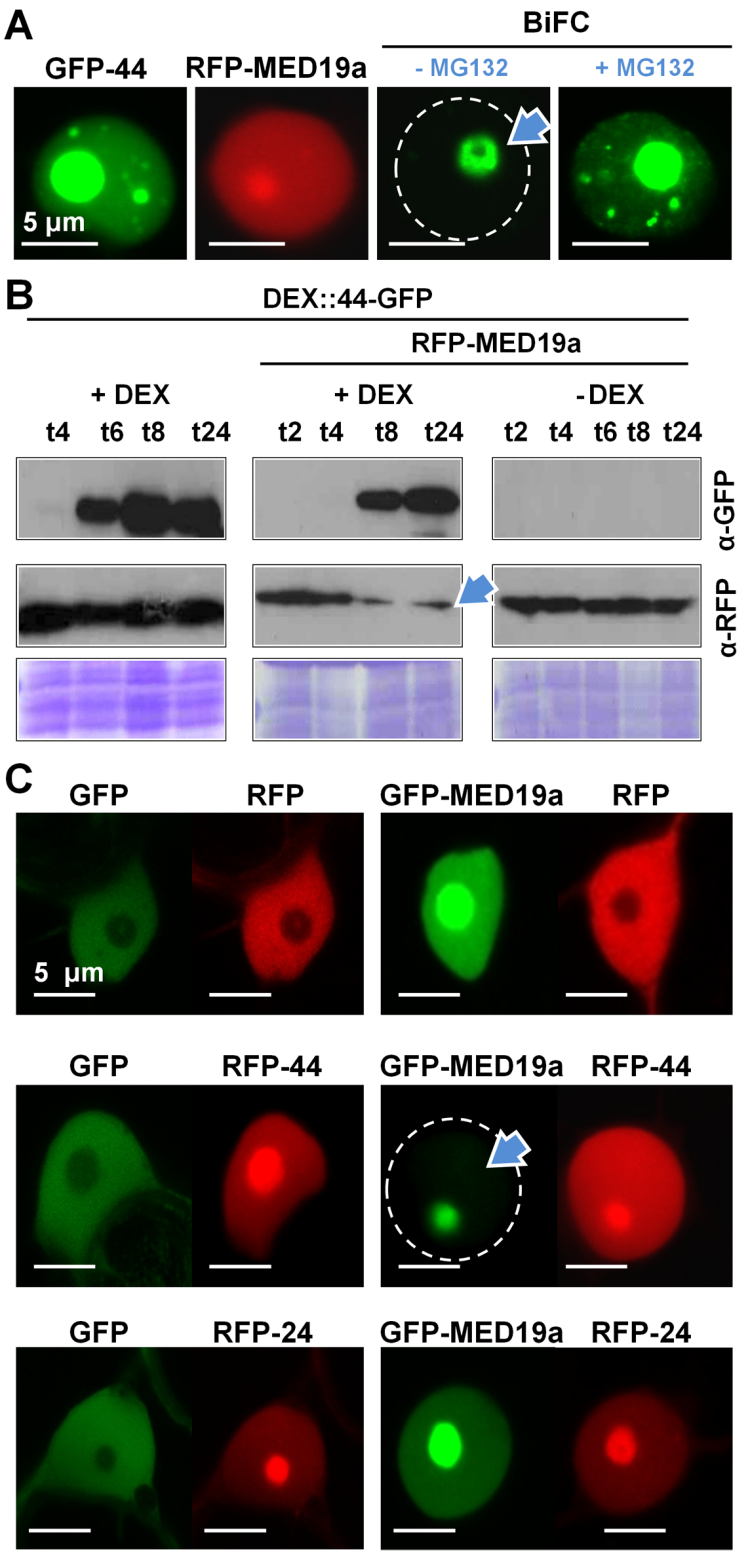
HaRxL44 destabilizes MED19a *in planta.* (A) Subcellular localisation of GFP-HaRxL44 (in green), RFP-MED19a (in red), and YFPc-HaRxL44 + YFPn-MED19a (BiFC, yellow) obtained by transient expression in *N. benthamiana*. n, nucleus. (B) Western blot analysis of protein extracted after transient expression of DEX::HaRxL44-GFP with RFP or RFP-MED19a in the presence or not of dexamethazone (DEX). Note the decrease in the level of MED19a observed in the presence of HaRxL44. (D) Co-localisation analysis between GFP-MED19a and nuclear-HaRxLs determined by transient assay in *N. benthamiana*. Note the lack of GFP-MED19a in the presence of RFP-HaRxL44 (arrow).

We monitored the subcellular location of RFP-MED19a and GFP-HaRxL44 using confocal microscopy. Both proteins localise to the nucleoplasm and nucleolus, whereas Bimolecular Fluorescence Complementation (BiFC) signals resulting from the co-expression of YFPc-MED19a and YFPn-HaRxL44 constructs are restricted to the nucleolus, following transient expression in *N. benthamiana.* No BiFC signal was detected in the nucleoplasm, the site of Mediator function (blue arrow, Figure 3A). The destabilisation of RFP-MED19a in the presence of GFP-HaRxL44 was quantifiable by both Western blotting (blue arrow, Figure 3B, Figure S5A) and confocal microscopy (blue arrow, Figure 3C). Furthermore, no decrease in the amount of GFP-MED19a was observed in coexpression experiments with RFP-24 and RFP-45 constructs, which encode other nuclear HaRxLs (Figure 3C, Figure S5A), suggesting that MED19a is specifically targeted by the HaRxL44 effector. As *MED19a* transcript levels were not affected in HaRxL44 lines (Figure S5B), we conclude that HaRxL44 destabilizes MED19a at the protein level. Taken together these results show that MED19a, which is found in both nucleoplasm and nucleolus, disappears in the nucleoplasm in the presence of HaRxL44, and perhaps persists in the nucleolus because of low proteasome activity in the nucleolus. Since Mediator is known to function in the nucleoplasm, this HaRxL44-mediated degradation of MED19 likely affects Mediator activity.

**Figure 4:**
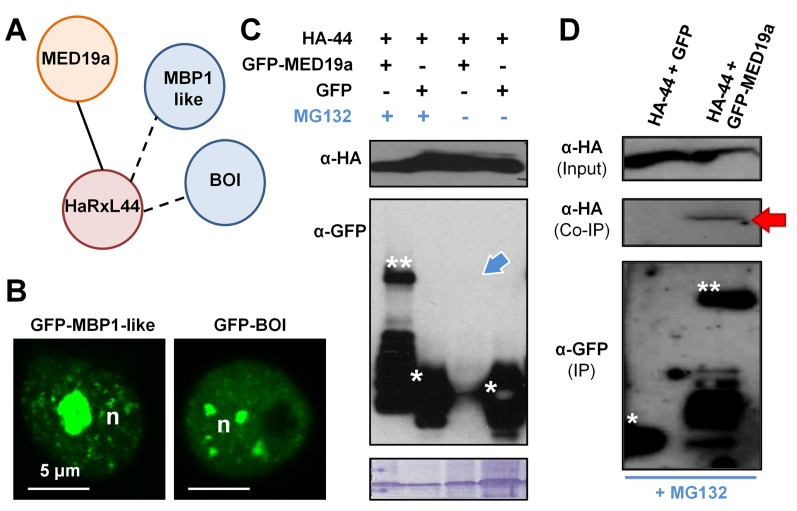
HaRxL44 interacts with and destabilizes MED19a in a Proteasome-dependant manner. (A) Schematic representation of the relevant interactions obtained by Y2H between HaRxL44 and *Arabidopsis* cDNA library. Data extracted from Mukhtar et al. (2011) [17]. (B) Subcellular localisation of GFP-BOI and GFP-MBR1–like determined by transient expression in *N. benthamiana.* (C) Immunoblotting of protein extracted from *N. benthamiana* leaves after transient assay, in presence or not of MG132 for 4 h. (D) Immunoblotting of protein extracted from *N. benthamiana* leaves after transient assay. Note the co-immunoprecipitation (Co-IP) of HA-HaRxL44 with GFP-MED19a in the presence of proteasome inhibitor.

In the Y2H screen [17], HaRxL44 was found to interact with two E3 ligases (Figures 4A and S3), BOTRYTIS SUSCEPTIBLE 1 (BOI; AT4G19700) and MED25-BINDING RING-H2 PROTEIN-like (MBR1-like; AT1G17970). We investigated whether these E3 ligases are present in the same plant cell compartment as HaRxL44 and MED19a. We investigated the subcellular distribution of these two E3 ligases, by transiently expressing GFP-tagged versions of BOI and MBR1-like in *N. benthamiana* (Figure 4B). GFP-BOI localises to the nucleoplasm and accumulates in foci, in four to five large bodies. Furthermore, no GFP-BOI signal was detected in the plant cell nucleolus (Figure 4B). GFP-MBR1–like was also localised to the plant cell nucleus (Figure 4B), in a pattern similar to that observed for proteins involved in RNA splicing [41]. GFP-MBR1–like accumulated in large amounts in the plant cell nucleolus and had a punctate distribution in the nucleoplasm (Figure 4B). In order to test whether one of the two E3-ligases interacting with HaRxL44 in Y2H might be responsible for MED19a degradation, we tested the phenotype of *BOI* and *MBR1-like* loss-of-function mutants during *Hpa* infection. Surprisingly, both the *boi* RNAi line and the *mbr1-like* T-DNA KO line were more susceptible to *Hpa* (Figure S5C). However, such loss-of-function experiments are difficult to interpret because BOI and MBR1-like might also affect other components of the plant immune system, leading to an increase in plant susceptibility.

As HaRxL44 interacts in Y2H analysis with E3 ligases located in the plant cell nucleus (Figures 4 and S3), we hypothesised that HaRxL44 acts as an adaptor protein for E3 ligases, mediating the degradation of MED19a. Indeed, we showed that inhibition of the proteasome by the addition of 100 µM MG132 for 4 h prevented HaRxL44-induced degradation of GFP-MED19a (Figure 4C). The addition of 100 µM MG132 during protein extraction prevented the degradation of GFP-MED19a in the presence of HA-HaRxL44 and made it possible to confirm the interaction of these proteins *in planta*, by co-immunoprecipitation (red arrow, Figure 4D). We next tested if blocking the proteasome would allow the detection of the interaction between HaRxL44 and MED19a in the nucleoplasm. We showed that addition of MG132 1 h before observation with confocal microscopy allowed the detection of the interaction between YFPc-MED19a and YFPn-HaRxL44 in the nucleoplasm by BiFC (Figure 3A). Thus, HaRxL44 interacts with MED19a, a positive regulator of plant immunity to *Hpa*, leading to its destabilisation in a proteasome-dependent manner.

**Figure 5:**
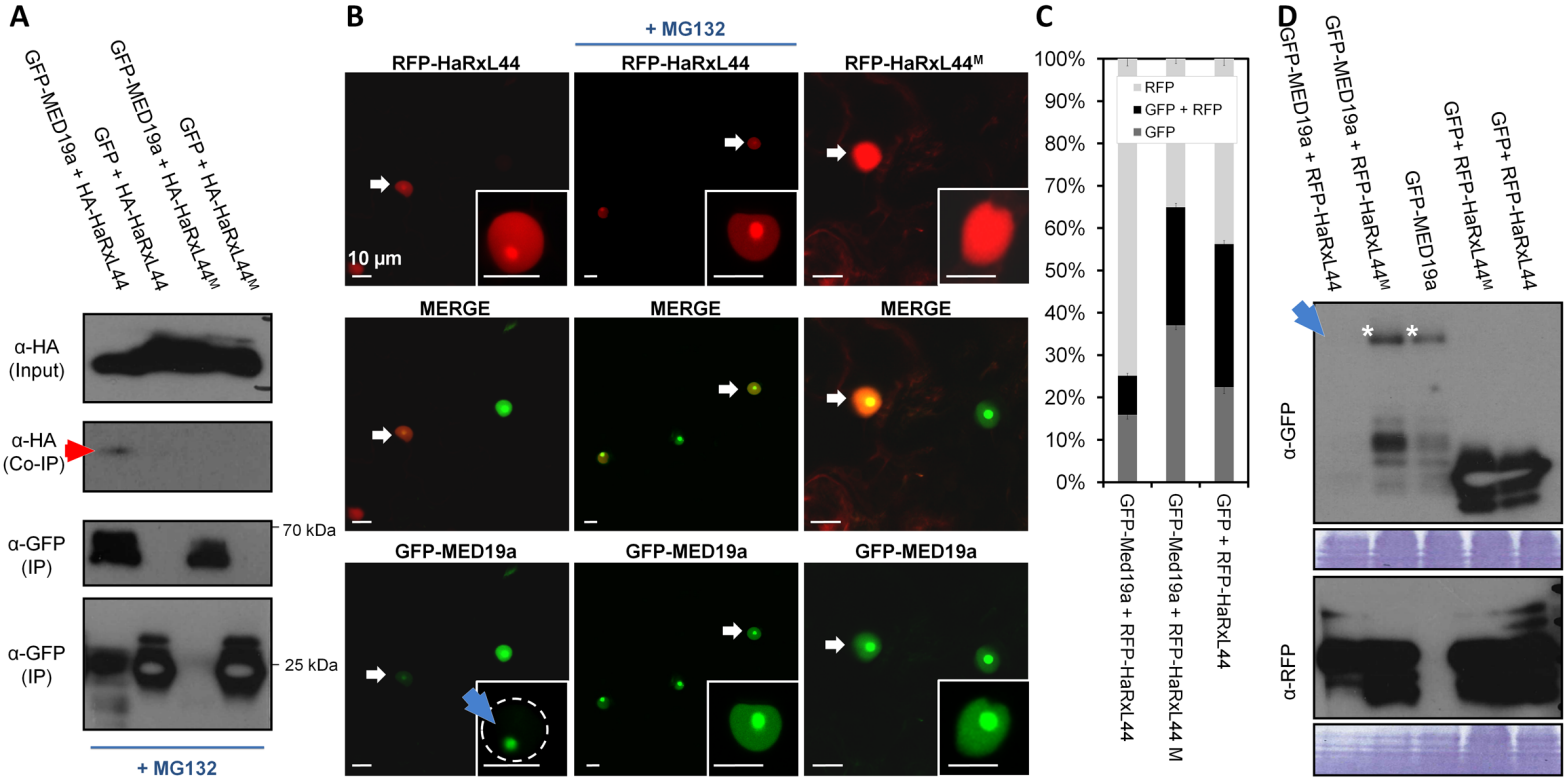
Interaction between MED19a and HaRxL44 is important for HaRxL44–induced MED19a degradation via proteasome. (A) Immunoblotting of proteins, extracted from *N. benthamiana* leaves after transient assay. Note the absence of Co-IP of HA-HaRxL44^M^ with GFP-MED19a. (B) Co-localisation analysis between GFP-MED19a and RFP-HaRxL44 or HaRxL44^M^ determined by transient assay in *N. benthamiana*. Note the lack of GFP-MED19a in the presence of RFP-HaRxL44 (arrow) but not HaRxL44^M^. (C) Quantification of the number of fluorescent nucleoplasm observed in nucleus transformed with GFP-MED19a in the presence or not of RFP-HaRxL44 or RFP-HaRxL44^M^. All the confocal pictures were taken with PMT 1 (494–541 nm) at Gain: 864 and PMT 2 (591–649 nm) at Gain: 844. Note the decrease in GFP-MED19a transformed cells in the presence of RFP-HaRxL44 in comparison with RFP alone or RFP-HaRxL44^M^.

In order to check if the interaction between HaRxL44 with MED19a is important for its degradation, we generated a series of HaRxL44 mutants by NAAIRS-scanning mutagenesis [42]. We obtained one mutant, HaRxL44^M^, mutated in the nucleolus-localization signal (Figure S6), which no longer interacts with MED19a by Co-IP when transiently expressed in *N. benthamiana* (Figure 5A). In contrast with HaRxL44, which is visible in the nucleoplasm and the nucleolus (Figure 5B), HaRxL44^M^ presents a nuclear-cytoplasmic localisation (Figure 5B). Using both cell biology (Figure 5B, 5C) and biochemistry (Figure 5D) we showed that HaRxL44^M^ no longer degrades MED19a when transiently co-expressed *in planta*. Thus, the interaction between HaRxL44 and MED19a is important for proteasome-dependent MED19a degradation.

First, we verified the degradation of MED19a in the presence of HaRxL44 in Arabidopsis, by generating a transgenic line expressing both GFP-MED19a and 3HA-Strep2-HaRxL44 (or 3HA-Strep2-GUS as control). We showed that, as we observed in *N. benthamiana*, MED19a is degraded in the presence of HaRxL44 in *Arabidopsis* and the addition of MG132 blocks the effect of HaRxL44 on MED19a stability (Figure S5D). We then investigated whether the presence of HaRxL44 affects the interaction between MED19a and the Mediator complex. We found that, even in the presence of 3HA-HaRxL44, MED6 co-immunoprecipitates with GFP-MED19a in *Arabidopsis* (Figure S5E), suggesting that MED6 and GFP-MED19a also associate in the nucleolus. However, overproduction of MED19a and HaRxL44 in *Arabidopsis* may affect the stoichiometry or nuclear/nucleolar distribution of interactions between MED19a and the Mediator complex, or Mediator subcomplexes, obscuring potential effects of HaRxL44 on the integration or stability of MED19a subunits in the Mediator complex.

### JA/ET Signalling Is Induced in the Presence of HaRxL44, the Absence of MED19a, and 3 d After *Hpa* Infection

**Figure 6:**
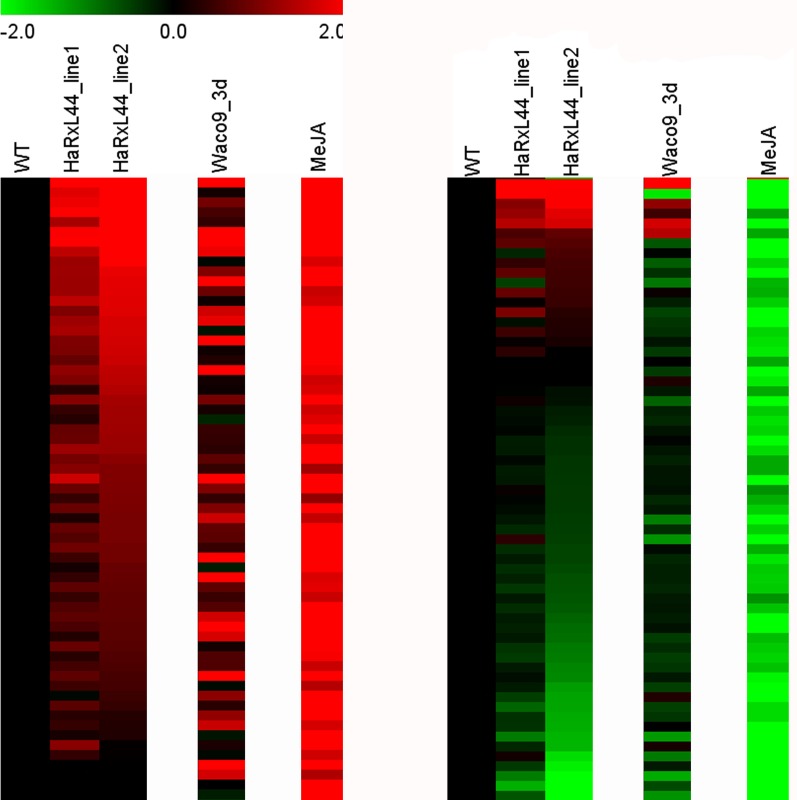
HaRxL44 expression affects JA/ET-regulated gene expressions. Expression of the JA/ET-regulated gene reported by Jung et al. (2007) [43] in HaRxL44 lines and 3 DAI with *Hpa* Waco9.

**Figure 7:**
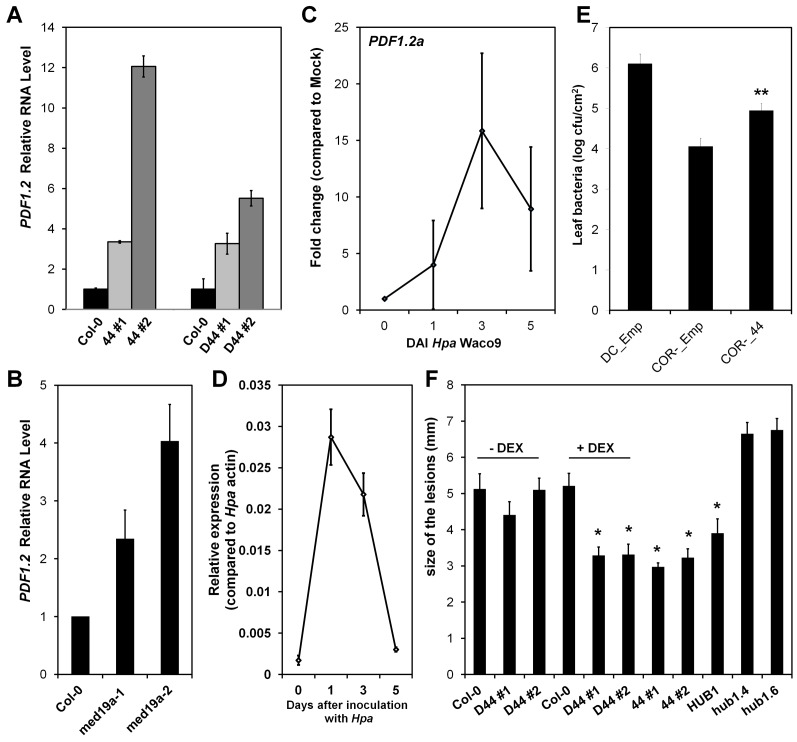
HaRxL44-expressing lines, *med19a* mutants show elevated JA/ET signalling, which is also observed after *Hpa* infection. (A and B) qRT-PCR results on *PDF1.2* marker gene. Data are presented as average fold induction compared with control of three biological replicates ± SD. (C) Expression pattern of *PDF1.2* during a time course of *Hpa* Waco9 infections in *Arabidopsis* Col-0 extracted from expression profiling experiment. (D) Expression pattern of *HaRxL44* during a time course of *Hpa* Waco9 infection in *Arabidopsis* Col-0 analysed by qRT-PCR. Data are presented as average fold induction compared with control of three biological replicates ± SD. (E) Monitoring of *Pst* growth in *Arabidopsis* Col-0. DC_Emp, *Pst* DC3000 strain carrying EDV vector; COR-_Emp, *Pst^COR^* ^−^ strain carrying EDV vector, COR-_44, *Pst^COR^* ^−^ strain carrying EDV-HaRxL44. Error bars represent the standard error of the mean (Tukey–Kramer test, *p* value<0.01). (F) Monitoring of *Botrytis cinerea* growth 5 DAI in transgenic lines expressing HaRxL44 under the control of DEX inducible promoter (D44 lines) in the presence or not of dexamethazone and under the control of 35S promoter (44 lines). Col-0, HUB1 OE, as well as *hub1* KO mutants were used as controls. Error bars represent the standard error of the mean. Asterisks represent the significance of individual unpaired *t* tests comparing the given column with the control (*p* value<0.01).

As MED19a is part of a major transcriptional regulatory complex, we then investigated whether and how *HaRxL44* expression affects transcription. Illumina RNA-sequencing revealed a positive correlation between the genes differentially up-regulated in HaRxL44-lines and by methyl JA (MeJA) treatment [43] (Hypergeometric probability <0.001; Figure 6, Tables S1 and S2). No correlation was observed for down-regulated genes in HaRxL44–line 1 (Hypergeometric probability = 0.98). This result can be explained by the lower number of genes differentially expressed in HaRxL44-line 1 compared to HaRxL44–line 2. However, the average fold change in HaRxL44–line 1 is still correlated to what is observed in HaRxL44–line 2 (Figure 6, Table S2). We confirmed, by QRT-PCR, that JA/ET marker genes (*PDF1.2*, *JAZ1*, and *JAR1*) were induced in HaRxL44-lines and in *med19a* mutants, with respect to WT levels (Figures 7A, 7B and S7A, S7B). Two of the five JA-responsive genes from the JA biosynthesis pathway [44], *OPR3* (AT2G06050) and *LOX2* (AT3G45140), were up-regulated (Figure S7C, Table S2), suggesting that HaRxL44 may induce JA/ET signalling.

We then checked whether the induction of JA/ET-responsive genes in the presence of HaRxL44 was biologically significant. We conducted gene expression profiling over a time course of *Hpa* infection in *Arabidopsis* and found that *PDF1.2* induction is observed 3 d after infection (DAI), when *HaRxL44* transcription was induced (Figure 7C, 7D). Furthermore, the induction of JA/ET-responsive genes in HaRxL44 transgenic lines was similar to the induction observed during early stages of *Hpa* infection in susceptible accessions of *Arabidopsis* (Figure 6, Tables S1 and S2). Thus, JA/ET signalling is induced in the presence of HaRxL44, the absence of MED19a, and 3 d after *Hpa* infection.

In *Pst*, the phytotoxin coronatine (COR) acts as an analogue of JA and contributes to bacterial invasion [45]. COR biosynthetic (COR^−^) mutants of *Pst* strain DC3000 exhibit reduced virulence on Arabidopsis when surface-inoculated [45]. In order to test if HaRxL44 was able to complement *Pst^COR^* ^−^ strain, we delivered HaRxL44 *in planta* by using EDV system (EDV-HaRxL44, [13]). When spray-inoculated in Arabidopsis Col-0 plants, *Pst^COR^* ^−^ growth was reduced by two logs (cfu/cm^2^) compared to *Pst* (Figure 7E). *Pst^COR^* ^−^ EDV-HaRxL44 growth was increased by one log compared to *Pst^COR^* ^−^ (**p value<0.01, Figure 7E). These results indicate that HaRxL44 is able to complement the deficiency of COR production in *Pst^COR^* ^−^ and supports a key role for this *Hpa* effector in the activation of the JA/ET pathway.

Because JA/ET-induced defence is effective against necrotrophs [3], we next challenged the transgenic lines expressing HaRxL44 with *Botrytis cinerea* (Figure 7F). As control, we used loss-of-function alleles of HISTONE MONOUBIQUITINATION1 (HUB1) shown to increase susceptibility to *B. cinerea*, and HUB1-OE lines that confer resistance to *B. cinerea* [34]. We observed that *B. cinerea* grew less well in HaRxL44-OE lines than in the WT, as also observed for HUB1-OE lines (*p value<0.01; Figure 7F, Figure S7D to S7F). Altogether, these results suggest that JA/ET-dependent defence is promoted in lines that express HaRxL44.

The Mediator complex is known to be important for JA/ET signalling [46]. In particular, MED25 and MED21 are key components of Mediator that regulate JA/ET-induced gene expression [34],[35]. We then tested if the *med21* and *med25* loss-of-function mutants are altered in *Hpa* growth. We observed that in both *med21* RNAi line and *med25* knock out (KO) mutants, *Hpa* growth was reduced compared with WT (Figure 2H). Thus, JA/ET-responsive gene transcriptional activation via Mediator is important for *Hpa* virulence.

### HaRxL44 Production, *MED19a* Gene Mutation, and *Hpa* Infection Suppress *PR1* Induction

**Figure 8:**
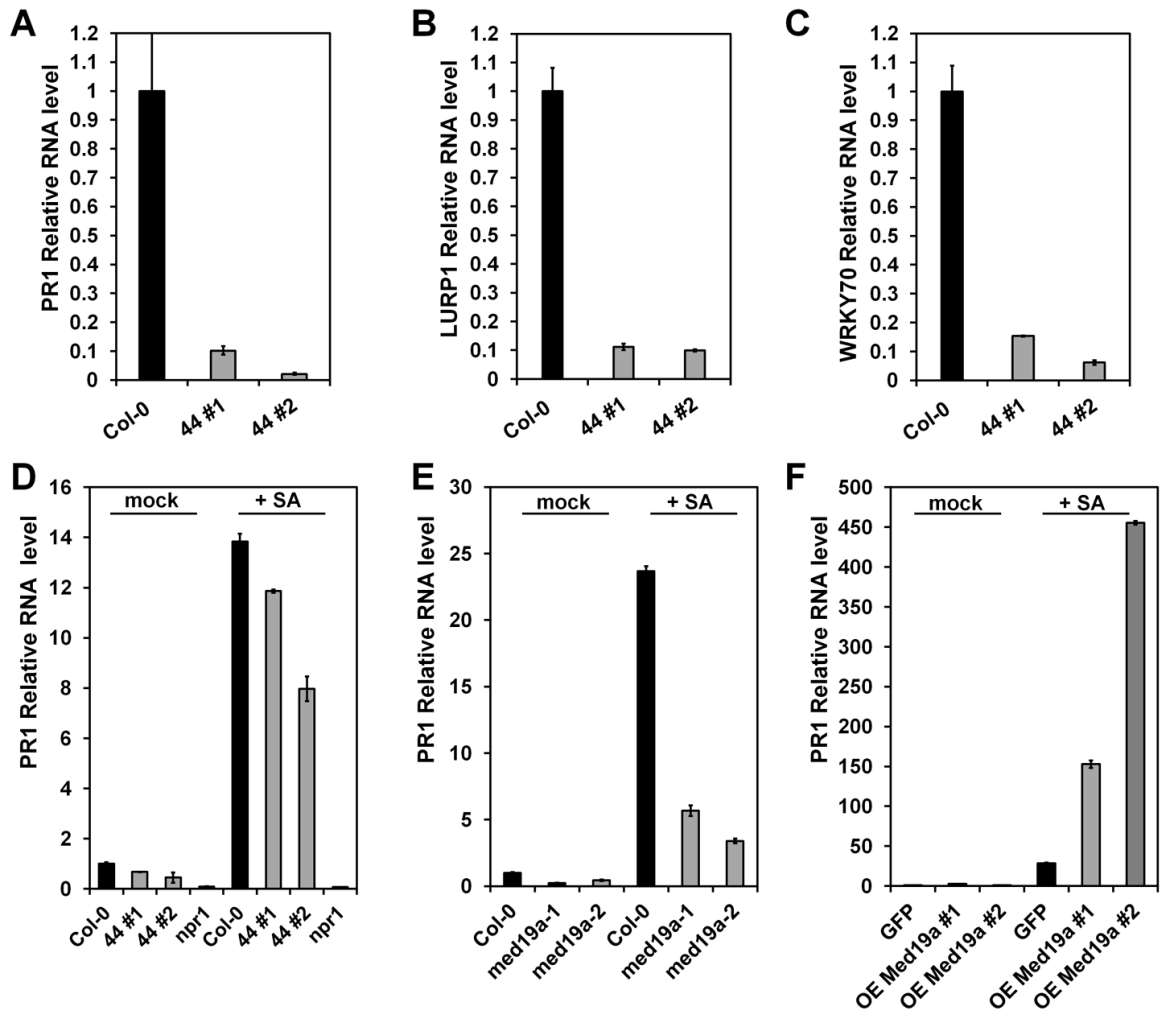
HaRxL44 expression, MED19a mutation suppresses *PR1* induction. (A–C) qRT-PCR on SA marker genes from 5-wk-old *Arabidopsis* plants. (D–F) qRT-PCR on *PR1* marker gene 8 h after SA treatment (200 µM) from 5-wk-old *Arabidopsis* plants. Data are presented as average fold induction compared with control of three biological replicates ± SD.

**Figure 9:**
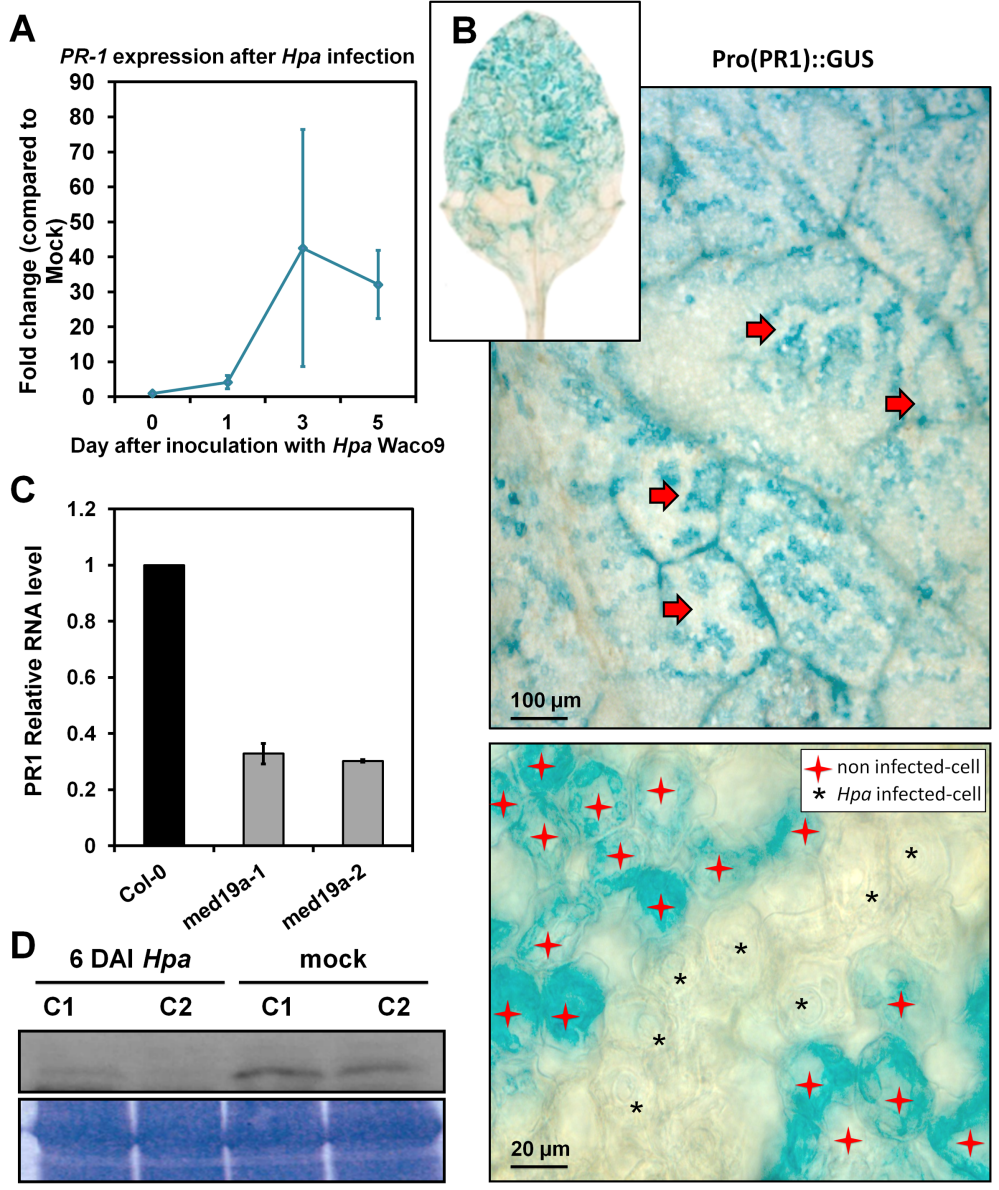
*Hpa* suppresses PR1 induction in infected cells. (A) qRT-PCR on *PR1* gene during a time course of infection of *Hpa* Waco9 in *Arabidopsis* Col-0. Data are presented as average fold induction compared with control of three biological replicates ± SD. (B) GUS staining of pro(*PR1*)::GUS in *Arabidopsis* leaves 6 DAI *Hpa* Waco9. Red arrows indicate *Hpa* hyphae's print surrounded by GUS stained cells. Note that no GUS was detected in *Hpa*-haustoriated mesophyll cell (black stars), while GUS staining was restricted to nonhaustoriated mesophyll cells (red stars). (C) qRT-PCR on *PR1* gene 6 DAI *Hpa* Waco9 in *Arabidopsis* Col-0, *med19a-1*, and *med19a-2* KO mutants. (D) Western blot on proteins extracted from *med19a* mutant complemented with GFP-MED19a after *Hpa* infection in comparison with mock treatment, using GFP antibody.

As the activation of the JA/ET defence pathway can antagonise SATI [3], we next assessed whether HaRxL44 suppresses SATI. We first observed, by QRT-PCR, that SA marker genes (*PR1*, *LURP1*, *WRKY70*, *PR2*, *PR5* genes) are down-regulated in HaRxL44 transgenic lines (Figures 8A–C and S8A, S8B). We then assessed *PR1* induction after elicitation. In HaRxL44 transgenic lines, basal *PR1* transcript levels are lower than those in the WT, resulting in a reduction of *PR1* induction levels 8 h after SA treatment (Figure 8D). Similar results were observed in *med19a* mutants (Figure 8E), whereas MED19a OE led to stronger *PR1* induction (from 5 to 15 times higher level of PR1 expression in MED19 OE lines compared to control plants; Figure 8F). We then investigated whether *Hpa* suppresses SATI. Expression profiling in Col-0 plant infected with *Hpa* Waco9 revealed a 40-fold change in *PR1* gene induction 3 DAI (Figure 9A). We then investigated the cell-specific expression pattern of *PR1*, by infecting *PR1*::GUS lines with *Hpa*. *PR1*::GUS staining was restricted to the plant vascular tissues in contact with *Hpa* 3 DAI (Figure S8C, S8D), whereas strong GUS staining was observed throughout the entire leaf 6 DAI (Figure 9B). An analysis of *PR1* expression patterns at the cellular level showed that *PR1*::GUS staining was absent from *Hpa*-infected cells, whereas *PR1*::GUS staining was observed only in the cell layer surrounding the mesophyll cells into which haustoria had penetrated (Figure 9B). Thus, *Hpa* suppresses SATI specifically in the haustoria-containing mesophyll cells to which the effector proteins are delivered. As expected, the amount of *PR1* mRNA generated in response to *Hpa* was lower in the absence of *med19a*, as shown by QRT-PCR (Figure 9C).

We next tested whether MED19a is degraded upon infection by *Hpa*. In GFP-MED19a lines, we tried to image signal in an infected mesophyll cell and compare this to the signal level to the signal in the neighbouring cells. However, measurement of fluorescence by confocal microscopy in deep tissues was too difficult to allow us to obtain reliable results. Therefore, we used the *med19a* mutant lines complemented with GFP-MED19a in order to check by Western blot analysis the GFP-MED19a protein level in *Hpa*-infected tissues compared to uninfected tissues. GFP-MED19a signal in infected tissues was reduced compared to uninfected tissues (Figure 9D), confirming that this positive regulator of plant immunity against *Hpa* is degraded after infection. We suggest that the destabilisation of MED19a by HaRxL44 results in transcriptional reprogramming, leading to changes in the balance between the JA/ET and SA pathways, promoting biotrophy.

## Discussion

We report here the functional analysis of an *Hpa* nonpolymorphic effector, HaRxL44. We verified Y2H data suggesting that HaRxL44 interacts with MED19a. We found MED19 to be a positive regulator of plant immunity against *Hpa*, leading to proteasome-dependent degradation of MED19a. Expression profiling reveals that JA/ET signalling is elevated in the presence of HaRxL44, in *med19a* knock-out mutants, and 3 d after *Hpa* infection. Strong JA/ET signalling is associated with weak SATI in both *Arabidopsis* plants expressing HaRxL44 and in *med19a* KO mutants, whereas strong SATI is observed in plants overexpressing *MED19a*. We confirmed that *Hpa* represses *PR1* expression specifically in the cells containing haustoria, into which RxLR effectors are delivered. Thus, HaRxL44 hijacks nondefensive aspects of the JA/ET signalling pathway, at the transcriptional level, via MED19a, resulting in reduced capacity to defend against biotrophs. A translocated chorismate mutase from *Ustilago maydis* was reported to be able to lower SATI by acting on SA biosynthesis [5]. In contrast, we report here a new mechanism of SATI suppression by means of a biotrophic oomycete effector that alters SA-dependent transcription by promoting degradation of MED19a, a transcriptional component involved in SA/JA crosstalk.

### MED19a, a Subunit of the Mediator Complex, Is a Positive Regulator of Plant Immunity to *Hpa*

*MED19*/*Rox3* was originally identified in a search for mutants increasing aerobic expression of the *CYC7* gene in yeast [47]. The nuclear localisation of this protein and the nonviability of null mutants suggest that the MED19/Rox3 protein is a general regulatory factor [47]. The purification of Mediator from a strain lacking the MED19 subunit [48] led to the demonstration that MED19/Rox3 regulated intermodule interactions in the *S. cerevisiae* Mediator complex. In *Arabidopsis*, MED19 is encoded by two genes, *MED19a* and *MED19b*. Only MED19a has been reported to be involved in Mediator complex formation in *Arabidopsis* [18]. HaRxL44 interacts with both MED19a and MED19b in Y2H screen [17]. We therefore tried to amplify both genes, but were unable to amplify the *MED19b* gene from cDNA or genomic DNA. Furthermore, no T-DNA insertion into the MED19b gene is available, limiting analyses of the function of this gene in response to *Hpa*. In this study, we focused on the role of MED19a during *Hpa* infection. However, it should be borne in mind that the phenotype observed for *med19a* KO mutants may be only partial, because MED19a and MED19b could have redundant functions. In *Arabidopsis*, there are other Mediator subunits encoded by several genes, such as MED10, MED20, MED22, and MED33. Transcript profiling with *med20a* and the RNA polymerase II subunit RPB2 mutant *nrpb2-3* revealed a high degree of overlap in the lists of genes displaying down-regulation in the two mutants [29]. This suggests that even a single mutation in one of several paralogs encoding an *Arabidopsis* Mediator subunit can lead to a quantifiable phenotype.

We first confirmed that MED19a was part of the Mediator complex, by demonstrating its interaction with MED6 and MED7 *in planta*. We then investigated the subcellular distribution of MED19a, which was found to be localised to the plant cell nucleoplasm, as reported for MED16 [26]. MED19a was also localised to the plant nucleolus. This is surprising, because Mediator is thought to associate with RNA polymerase II in the nucleoplasm. It has been suggested that Mediator regulates the action of other plant RNA polymerases [49]. The similarities between RNA polymerases II, IV, and V raise the possibility that Mediator may associate with another polymerase, either polymerase IV or polymerase V [49]. MED19a may even associate with the nucleolar RNA polymerase I or III. Indeed, Mediator subunits have been shown to interact with RNA polymerase I subunits in Y2H assays ([50], Figure S1). However, as the evidence concerning the possible role(s) of Mediator in directing the activity of other RNA polymerases remains inconclusive, we decided to focus on the role of MED19a in the regulation of transcription by RNA polymerase II. A proteomic analysis of human nucleoli revealed the presence of a large number of proteins with no known nucleolar function [51]. Nucleolar protein composition is not static and may undergo significant modification in response to the metabolic state of the cell [52]. The regulation of protein activity by nucleolar sequestration has been reported before [53],[54]. Indeed, this phenomenon has already been reported for human MED1 [55]. MED1 is phosphorylated by MAPK1 or MAPK3 during the G2/M phase, enhancing protein stability and promoting the entry of this molecule into the nucleolus [55]. We can speculate that MED19a is sequestered in the nucleolus to remove it from the functional pool of MED19a in the nucleoplasm.

We then investigated whether the presence of HaRxL44 affected the interaction between MED19a and the Mediator complex. We showed that even in the presence of HaRxL44, MED19a associated with MED6 in *Arabidopsis*. However, we cannot exclude the possibility that the overproduction of MED19a and HaRxL44 in *Arabidopsis* affects the stoichiometry between MED19a and the Mediator complex, obscuring potential effects of HaRxL44 on the integration of MED19a subunits into the Mediator complex.

### HaRxL44 Promotes the Proteasome-Dependent Turnover of MED19a

We show here that HaRxL44 interferes with Mediator function by promoting the proteasome-dependent degradation of MED19a. Effectors from plant pathogens have been reported to suppress various layers of plant defence by controlling the ubiquitination and degradation of proteins important for plant immunity via the proteasome.

AvrPtoB is a well-studied *Pseudomonas* syringae effector that mimics a plant E3 ligase [56] and facilitates the degradation of key components of PAMP-triggered immunity [57]–[60]. The *Xanthomonas* effector XopL has been shown to display E3 ubiquitin ligase activity *in vitro* and *in planta*, to induce plant cell death, and to suppress plant immunity [61]. The structural fold of the E3 ubiquitin ligase domain in XopL is unique, and the lack of cysteine residues in the XL-box suggests a noncatalytic mechanism for XopL-mediated ubiquitination [61]. The *P. syringae* effector HopM1 mediates the degradation, by the proteasome, of AtMIN7, a plant protein involved in the vesicular trafficking of defence components [62],[63]. Unlike AvrPtoB and XopL, HopM1 has no E3 ligase activity, suggesting that this effector acts as an adaptor protein, connecting AtMIN7 and the proteasome [62]. Several ubiquitin proteins have been identified in the *Meloidogyne incognita* secretome, and a ubiquitin extension protein secreted from the dorsal pharyngeal gland of *Heterodera schachtii* has also been detected [64],[65]. The *Magnaporthe oryzae* effector AvrPiz-t was recently reported to interact with a RING E3 ubiquitin ligase, APIP6, abolishing its ubiquitin ligase activity [66]. In addition, the *P. infestans* RXLR effector AVR3a has been shown to target and stabilise the nucleolar E3 ligase CMPG1, which is required for the programmed cell death triggered by the elicitin INF1 [67],[68]. However, the targets for the ubiquitination of these E3 ligases have yet to be determined.

We show here that HaRxL44 interacts with MED19a, destabilising this Mediator subunit in a proteasome-dependent manner. As HaRxL44 displays no sequence similarity to plant E3 ligases, we suggest that, like HopM1, HaRxL44 acts as an adaptor, presenting MED19a to the proteasome or to an E3 ligase. However, the mechanism by which HaRxL44 induces the degradation of MED19a remains unclear. Y2H screens have shown that HaRxL44 interacts with two E3 ligases: BOI and MBR1-like [17]. BOI is encoded by a gene from a multigene family with four known members, including BOI-RELATED GENE [69]. BOI was identified in a screen for proteins interacting with *BOTRYTIS SUSCEPTIBLE 1* (BOS1), which encodes an R2R3 MYB transcription factor involved in restricting necrotroph-induced necrosis [70]. BOI is an important player in plant immunity to necrotrophic pathogens [71]. BOI ubiquitinates BOS1, leading to its rapid degradation by the proteasome [71]. In addition to its role in restricting necrosis, BOI may integrate plant responses to diverse signals [72]. Indeed, Park et al. (2013) recently showed that BOI and DELLA proteins inhibit GA responses by interacting with each other, binding to the same promoters of GA-responsive genes, and repressing these genes. In the Y2H screen carried out by Mukhtar et al. (2011) [17], BOI was found to interact with four nuclear effectors from *Hpa*: HaRxL44, HaRxL10, ATR1, and ATR13. Thus, *Hpa* effectors may act on BOI function, to render the plant more susceptible to biotrophic pathogens.

It is not clear whether the HaRxL44-mediated degradation of MED19a by the proteasome has a positive or negative impact on transcription. It is well known that one major way of regulating transcription is to couple the activity of transcription factors to their destruction by the proteasome [73]. This “transcription-coupled destruction” mechanism of activator action [74] must serve a functional purpose, such that, if blocked, repeated rounds of transcriptional activation cannot occur [73]. This “unstable when active” phenomenon is seen with many transcriptional regulators, including the Mediator subunit MED25 [75]. In *Arabidopsis*, MED25 is a highly unstable protein, degraded by the proteasome both *in vitro* and *in vivo* [75]. A blockade of proteasome activity prevents MED25 from inducing flowering [75]. Two E3 ubiquitin ligases, MBR1 and MBR2, have been shown to polyubiquitinate MED25 *in planta*, supporting the “transcription-coupled destruction” model for the regulation of MED25. MBR1 and MBR2 are part of a small cluster of E3 ligases in *Arabidopsis* [76]. HaRxL44 has been shown to interact with MBR1-Like in Y2H screens [17]. Thus, HaRxL44 may recruit different E3-ligases, to promote the destruction of MED19a, thereby promoting *Hpa* growth.

In metazoans, Mediator complex subunits are degraded upon cell differentiation [77]–[79]. This observation is consistent with the notion that subcomplexes of Mediator may display cell type–specific activity [78]. The degradation of some subunits helps to turn off the expression of a large portion of genes, whereas the retention of other subunits is required for the expression of a smaller, highly specific subset of genes [78],[80]. Based on our results, we hypothesise that HaRxL44 targets MED19a for degradation, to block the transcription of genes important for plant immunity (i.e., genes important for SA-dependent defence), whereas MED19a degradation allows the transcription of a small number of genes beneficial for *Hpa*, including JA/ET-induced genes.

### HaRxL44 Affects the Balance of JA/ET and SA Signalling at the Transcriptional Level to Promote Biotrophy

We showed that JA/ET signalling is induced in the presence of HaRxL44 (or the absence of MED19a). Expression profiling using Illumina RNA sequencing revealed a positive correlation between the genes differentially up-regulated in HaRxL44-lines and by MeJA treatment [43]. No correlation was observed for down-regulated genes in HaRxL44–line 1, but this result can be explained by the low number of genes differentially expressed in HaRxL44–line 1 compared to HaRxL44–line 2. However, the average fold change in HaRxL44–line 1 is still correlated to what is observed in HaRxL44–line 2. This result is consistent with the quantitatively different phenotypes observed in these transgenic lines, such as susceptibility to *Hpa* (Figure 1B, [13]), induction of *PDF1.2* (Figure 7A), and suppression of SA-responsive genes (Figure 8A-D). Thus, we believe that HaRxL44 affects JA/ET-regulated gene expression. Indeed, HaRxL44-expressing plants showing activation of JA/ET-responsive genes are more resistant to the necrotrophic pathogen *B. cinerea* for which JA/ET-dependent defence is required. Conversely, HaRxL44 expression (or the absence of MED19a) resulted in a loss of *PR1*-induction and higher rates of biotrophic pathogen growth. These results suggest that HaRxL44 affects the hormonal balance between JA/ET and SA, promoting biotrophy, by acting on the transcriptional machinery of the plant. *Hpa* infection also led to the expression of JA/ET-responsive genes, confirming the biological significance of the results obtained in the functional analysis of HaRxL44.

JA/ET and SA-dependent defences are known to be antagonistic. *Arabidopsis* mutants with impaired SA accumulation, such as *eds4*, *eds5*, and *pad4*, display high levels of *PDF1.2* expression in response to inducers of JA/ET-dependent gene expression [81],[82]. Convincing evidence for such an antagonistic effect has also been reported for NON-EXPRESSOR OF PATHOGENESIS-RELATED GENES1 (*NPR1*) [83]. *NPR1* is the key regulator of SAR, an important reaction in defence against pathogens. The *Arabidopsis npr1* mutant displays high levels of JA/ET-responsive gene transcript accumulation and of JA and ET accumulation in response to *P. syringae* infection, suggesting that *NPR1* is involved in the SA-mediated suppression of JA/ET signalling [83]. Reciprocally, an *mpk4* mutant has been shown to display constitutive SA-dependent gene expression and higher SA levels and enhanced resistance to biotrophic pathogens [84]. MPK4 up-regulates JA/ET-responsive genes and simultaneously suppresses SAR, placing MPK4 at the heart of the antagonistic interaction between JA/ET and SA [84],[85].

The role of the Mediator complex in JA/ET and SA-responsive gene expression has recently been investigated. MED25, MED21, and MED8 have been shown to be important for the activation of JA/ET-induced gene transcription [34],[35]. MED25 plays a major role in the JA-responsive gene transcription pathway, through its interaction with the transcription factor MYC2, which plays a key role in the activation of JA-induced gene expression [86]–[88]. MED25 regulates JA-dependent defence responses, conferring resistance to necrotrophic pathogens, and a *med25* mutant has been shown to be more susceptible than the WT to the hemibiotroph *Fusarium oxysporum* [89]. The effect of a *med8* mutation on the JA/ET-induced expression of *PDF1.2* is readily detectable only in *med8 med25* double mutants [35]. MED21 RNA interference lines are susceptible to both *B. cinerea* and *A. brassicicola* [34]. MED21 has been shown to interact with a RING E3 ligase, HISTONE MONOUBIQUITINATION1 (HUB1), increasing resistance to necrotrophs [34].

MED14, MED15, and MED16 were recently reported to up-regulate SAR in *Arabidopsis* [37]–[39],[90]. We show here that mutations of the gene encoding MED19a increase the basal level of JA/ET-responsive gene transcription and decrease the responsiveness of *PR1* gene expression to SA. The abolition of *PR1* expression or the absence of MED19a (or the presence of HaRxL44) was associated with faster growth of *Hpa* in *med19a* KO mutants. Thus, HaRxL44 targets a positive regulator of plant immunity to biotroph pathogens, thereby interfering with hormonal balance and promoting biotrophy.

### Cell Type–Specific Suppression of Host Defences in Haustorial Pathogens

When the first results from expression profiling host gene expression became available, a paradox emerged [91]. Even susceptible plants, in which *Hpa* is presumably suppressing host defences, show strong activation of a set of plant genes induced by SATI during SAR. Why does this defence activation not preclude pathogen infection? Our cell biology analysis reported here resolves this paradox. We show that, during *Hpa* infection, the pathogen blocks *PR1* induction in cells with haustoria, suggesting that the HaRxL effectors act at the transcriptional level, blocking *PR1* expression (and presumably other genes of the SATI regulon), to promote virulence. Further analysis requires methods, currently under development, to expression profile specifically from infected cells. HaRxL44 is unlikely to be the sole effector that accomplishes this shift in hormonal balance that promotes biotrophy. Indeed, other nuclear-HaRxLs have been shown to interact with the Mediator complex as well as with other regulators of JA/ET pathway, like JAZ proteins [17]. Functional analyses of these effectors should facilitate the discovery of new components of nuclear immunity and the engineering of improvements to plant defences, to strengthen disease resistance in crops.

## Methods

### Cloning of HaRxL44 and MED19a and Bioinformatics

To generate HaRxL44 constructs, primers were designed from the *Hpa* Emoy2 genome version 8.3. HaRxL44 was amplified from the signal peptide cleavage site (ΔSP-HaRxL44) until the stop codon using genomic DNA extracted from *Hpa* Emoy2 conidiospores, proof reading polymerase (Accuprime Pfx, Invitrogen), and standard PCR conditions. The HA tag sequence was added to the Fw primer (CACCATGTATCCGTACGACGTACCAGACTAC GCAATTGAAGTTGTCCCC) in order to create an HA-HaRxL44–tagged version. The PCR fragment was inserted into the pENTR-D-TOPO and then in the plant expression vectors pK7WGF2, dP2 [92], and pBAV150 using Gateway Technology (Invitrogen). The constructs were sequenced by The Genome Analysis Centre (Norwich, UK) and transformed into *Agrobacterium tumefaciens* strains GV3101 and GV3103.

For the prediction of HaRxL44 nucleolar localisation signal, NoD [93] was used http://www.compbio.dundee.ac.uk/www-nod/index.jsp. HaRxL44^M^ NAAIRS mutant was generated by overlapping PCR using the primers Fw AATGCTGCTATA CGATCGAAACACAAGAGG and Rev CGATCGTATAGCAGCATTCTTGTGCCAGCC.

MED19a (AT5G12230) was amplified from *Arabidopsis* Col-0 cDNA obtained from flowers using the primers: MED19a F1-CACCATGGAGCCTGAACGTTTAAA and MED19a R1-TTAGCCAGCAACCCTTATTGCACC. BOI was amplified from *Arabidopsis* Col-0 genomic DNA using the primers F2-CACCATGGCTGTTCAAGCTCATC ACATGAACATTTTC and R2-TCAAGAAGACATGTTAACATGCACACTAGCGTTCA TGACCATATCGC and MBR1-like (At1G17970) using the primers F3-CACCATGTCTTCTACAACAATCGGCGAGCACATCAG and R3-TTAAGGCTTGCC ATATGCTGCCTTCTTACAGACCG. The PCR fragment was inserted into the pENTR-D-TOPO and then in the plant expression vectors pK7WGF2 and pH7WGR2 using Gateway Technology (Invitrogen). The constructs were sequenced by The Genome Analysis Centre (Norwich, UK) and transformed into *A. tumefaciens* strain GV3103.

### Analysis and Isolation of Arabidopsis Mutants

To isolate homozygous *med19a-1/med19a-1* and *med19a-2/med19a-2* plants, we could not analyse the segregation of the kanamycin marker carried by the T-DNA on progenies because of the loss of kanamycin resistance in these SALK lines (SALK_037435.47.85x and SALK_034955.56.00x). For *mbr1-like* mutant, we use a homozygous line from the SALK named SALK_025248.37.45.x. T-DNA insertions were checked by PCR genotyping using T-DNA left border and gene-specific primers designed by the Salk Institute Genomic Analysis Laboratory (SIGnAL) (http://signal.salk.edu/tdnaprimers.2.html) using default conditions. Homozygote lines were identified.

### Protein Extraction, Co-Immunoprecipitation, and Western Blot

For protein extraction, frozen plant tissues were ground and mixed with an equal volume of cold protein isolation buffer [20 mM Tris-HCl (pH 7.5), 1 mM EDTA (pH 8.0), 5 mM DTT, 150 mM NaCl, 0.1% SDS, 10% glycerol, 1× Protease Inhibitor Cocktail (Sigma)]. The mixture was spun down, and the supernatant was transferred to a new tube and boiled in 5× SDS loading buffer [300 mm Tris-HCl (pH 6.8), 8.7% SDS, 5% *β*-mercaptoethanol, 30% glycerol and 0.12 mg/ml bromophenol blue].

For co-immunoprecipitation experiment, frozen leaf samples were ground in liquid nitrogen. The resulting powder was transferred into prechilled SM-24 20 mL centrifuge tubes containing chilled extraction buffer (4–10 mL) [1 M Tris HCl pH 7.5, 5 M NaCl, 0.5 M EDTA, 20% glycerol, 10 mM DTT, 1× Protease inhibitor (Sigma), 20% Triton X-100, 2% PVPP]. Tubes were vortexed and equilibrated before centrifugation 20 min at 20,000 rpm at 5°C. After centrifugation, supernatants were filtered to remove plant debris (Biorad Poly-Prep Chromatography columns). Proteins were quantified by Bradford assay. Three micrograms of total protein extracts were used for co-immunoprecipitation in protein Lo-Bind safe-lock tubes (Eppendorf) in which 25 µL of slurry solution of GFP beads (Chromotek) were added. Tubes were incubated on a rolling wheel for 2 to 4 h at 5°C. After incubation beads were washed with extraction buffer without PVPP by repeated low-speed centrifugations (up to four washes). Beads were resuspended in 5× SDS loading buffer *prior* to flash-freezing in liquid nitrogen.

Proteins were separated by SDS-PAGE, electro-blotted onto PVDF membrane (Biorad), and probed with horseradish peroxidase-conjugated anti-RFP (Abcam) or anti-GFP (Roche) antibody. MED6 and MED7 primary antibodies (from Bjorklund's lab) were used at 1∶1,000. Bands were visualized by chemiluminescence using Pico/Femto (Thermo Scientific).

### Pathogen Assays

For *Hpa* infection, 10-d-old plants were spray-inoculated to saturation with a spore suspension of 5.10^4^ spores/ml. Plants were kept in a growth cabinet at 16°C for 3 d with a 16 h photoperiod. To evaluate conidiospore production, 10 pools of 2 plants were harvested in 1 ml of water for each line. After vortexing, the amount of liberated spores was determined with a haemocytometer as described by [94]. Statistical analyses have been performed from three independent experiments, using ANOVA.

For *B. cinerea* infection, spores from the fungus strain B05.10 were obtained from Dr. Henk-Jan Schoonbeek (John Innes Centre, Norwich, UK). Inoculation of *Arabidopsis* with *B. cinerea* spores was performed as described previously [95]. Briefly, 5-wk-old plants were inoculated with a suspension of 2.5×10^5^ spores/mL in quarter-strength potato dextrose broth (6 g/L). Five-microliter droplets of spore suspension were deposited on six leaves per plant, with eight to 12 plants per experiment, and lesion diameters were measured at 3 d postinfection.

*Pst* infection was performed as described by [96]. Briefly, Arabidopsis plants were sprayed with bacterial suspensions carrying the EDV construct generated by Fabro et al. (2011) [13] (supplemented with 0.05% Silwet L-77). Plants were then covered with a transparent lid for 48 h. Infected leaf samples were collected at 4 DAI, ground in sterile 10 mM MgCl_2_, serially diluted, and spotted on NYG or low-salt LB (Luria-Bertani) agar medium containing appropriate antibiotics. Numbers of colonies were counted after 2 d of incubation at 28°C.

### SA-Induced *PR1* Expression Analysis

For SATI assay, 5-wk-old *Arabidopsis* plant were used. Leave disks were equilibrated in water in the dark overnight, and the solution was changed for 200 µM SA (Sigma) in the morning. After 8 h of incubation with SA or mock, leaf disks were quickly dried and flash-frozen in liquid nitrogen. About 20 leaf disks per condition were used for RNA extraction.

### Transient and Stable Gene Expression *in Planta*

For transient assay analysis, *A. tumefaciens* strains GV3101 and GV3103 were used to deliver respective transgenes in *N. benthamiana* leaves, using methods previously described [97]. Protein stability was assessed using Western blot, as described by [98]. For stable expression *in planta* of selected candidates, *Arabidopsis* WT (Col-0) plants were transformed using the dipping method [99]. Briefly, flowering *Arabidopsis* plants were dipped with *A. tumefaciens* carrying a plasmid of interest, and the seeds were harvested to select the T1 transformants on selective GM media. T1 plants were checked for expression of the construct of interest either by fluorescence microscopy and/or by Western blot analysis. T2 seeds were sown on selective GM media, and the proportion of resistant versus susceptible plants was counted in order to identify lines with single T-DNA insertion. Transformed plants were transferred to soil and seeds collected. For each construct, three independent transformed plants were analyzed. T3 homozygotes plants were used for *in vivo* confocal microscopy and pathotests.

### RNA Extraction, cDNA Synthesis, and qRT-PCR

Frozen plant tissues were ground to a fine powder in liquid nitrogen using a precooled pestle and mortar. The powder was immediately transferred to a 1.5 ml tube and rapidly frozen in liquid nitrogen. Batches of 12 samples were thawed on ice, and 1 ml Tri-Reagent (Sigma) was added to the tubes and incubated at room temperature for 10 min. The solution was centrifuged for 20 min at 12,000× g, and the supernatant was transferred to a clean tube containing an equal volume of isopropanol. The tube was incubated overnight at −20°C and centrifuged for 10 min at 12,000× g, 4°C. Pellets were washed with 70% ethanol, air dried, and resuspended in RNase-free water. The yield and integrity of the RNAs were assessed by measuring the optical density at 260 nm and 280 nm Micro-Volume UV-Vis Spectrophotometer for Nucleic Acid and Protein Quantitation (Nanodrop, Thermo Scientific, UK) and agarose gel.

Five micrograms of total RNAs were used for generating cDNAs in a 20 µl volume reaction according to Invitrogen Superscript II Reverse Transcriptase protocol. The obtained cDNAs were diluted five times, and 5 µl were used for 10 µl qPCR reaction, and 10 µl were used for 20 µl PCR reaction.

qPCR was performed in 20 µl final volume using 10 µl SYBR Green mix (Sigma), 10 µl diluted cDNAs, and primers. qPCR was run on the CFX96 Real-Time System C1000 thermal cycler (Biorad) using the following program: (1) 95°C, 4 min; (2) [95°C, 10 s, then 62°C, 15 s, then 72°C, 30 s]×40, 72°C, 10 min followed by a temperature gradient from 65°C to 95°C, and then 72°C, 10 min. The relative expression values were determined using EF1α (At5g60390) as reference gene and the comparative cycle threshold method (2^−ΔΔCt^). Primers were designed using Primer3 with the default settings.

For RNA sequencing, total RNAs were extracted using TRI reagent (Sigma) and 1-bromo-3-chloropropane (Sigma) according to the procedure of the manufacturer. RNAs were precipitated with half volume of isopropanol and half volume of high salt precipitation buffer (0.8 M sodium citrate and 1.2 M sodium chloride). RNA samples were treated with DNaseI (Roche) and purified by RNeasy Mini Kit (Qiagen) according to the procedure of the manufacturers.

### RNA Sequencing

RNA sequencing was performed as described by [100]. Briefly, total RNAs (3 µg) were used to generate first strand cDNAs using an oligo(dT) primer comprising P7 sequence of Illumina flow cells. Double-strand cDNAs were synthesised as described previously [101]. Purified cDNAs were subjected to Covaris shearing (parameters: intensity, 5; duty cycle, 20%; cycles/burst, 200; duration, 90 s). End repairing and A-tailing of sheared cDNAs were carried out as described by Illumina. Y-shaped adapters were ligated to A-tailed DNA and subjected to size selection on agarose gel. The gel-extracted library was PCR enriched and quantified using qPCR with previously sequenced similar size range Illumina libraries. The libraries were sequenced on Illumina Genome Analyzer II.

Illumina libraries were quality-filtered using FASTX Toolkit 0.0.13 with parameters −q20 and −p50 (http://hannonlab.cshl.edu/fastx_toolkit/index.html). Reads containing “N” were discarded, and read qualities were converted from Illumina fastq to Sanger fastq format. The libraries were separated using perfect match to the barcode. The sub-library was artefact-filtered using FASTX-toolkit. Quality-filtered libraries were aligned to the *Arabidopsis* Col-0 genome sequence (TAIR10) using Bowtie version 0.12.8 [102] and reads with up to 10 reportable alignments were selected. Unaligned reads from previous steps were used to align to transcript sequences of *Arabidopsis* Col-0 (ftp://ftp.Arabidopsis.org/home/tair/Sequences/blast_datasets/TAIR10_blastsets/TAIR10_cdna_20101214_updated) using Bowtie version 0.12.8. Linking of each sequenced read (Tag) to gene was carried out using the following considerations: reads aligning to each gene limits were assigned to that gene; reads aligning to genes with overlapping gene limits were split equally between them; and reads aligning to more than 10 genes were discarded. Differential expression analysis was performed using the R statistical language version 2.11.1 with the Bioconductor [103] package, edgeR version 1.6.15 [104] with the exact negative binomial test using tagwise dispersions.

### Microscopy

For co-localisation assays in *N. benthamiana*, cut leaf patches were mounted in water and analysed on a Leica DM6000B/TCS SP5 confocal microscope (Leica Microsystems) with the following excitation wavelengths: GFP, 488 nm; YFP, 488 nm; RFP, 561 nm. For *in vivo* localisation in *Arabidopsis*, 10-d-old *Hpa*-infected seedlings were mounted in water and analysed on a Leica DM6000B/TCS SP5 confocal microscope (Leica Microsystems) with the following excitation wavelengths: CFP, 458 nm; GFP, 488 nm; RFP, 561 nm.

GUS activity was assayed histochemically with 5-bromo-4-chloro-3-indolyl-β-d-glucuronic acid (1 mg/ml) in a buffer containing 100 mM Sodium Phosphate pH 7, 0.5 mM Potassium Ferrocyanide, 0.5 mM Potassium Ferricyanide, 10 mM EDTA, 0.1% Triton. *Arabidopsis* leaves were vacuum-infiltrated with staining solution and then incubated overnight at 37°C in the dark. Destaining was performed in 100% ethanol followed by incubation in chloral hydrate solution. Sections were observed with a Zeiss Axioplan 2 microscope (Jena, Germany).

Aniline blue staining was used to stain callose structures in plant tissues [105], which appeared after infection, like ring or encasements of *Hpa* haustoria, or like dots after *Pseudomonas* infection or PAMP treatment. Samples (either *Hpa*-infected seedlings or leaf disks punctured from PAMP/*Pseudomonas*-infiltrated leaves) were cleared in 100% methanol, washed in water, and then stained with aniline blue (0.05% w/v in 50 mM phosphate buffer pH 8) overnight. Samples were observed with a Leica DM6000B/TCS SP5 confocal microscope (Leica Microsystems).

## Supporting Information

Figure S1 **Interactomic data extracted from Mukhtar et al. (2011)** [**17**] **.** (A) List of the plant proteins interacting with HaRxL44 in Y2H. (B) Cytoscape representation of the network of the interactions obtained in Y2H for Mediator subunits. Data extracted from Mukhtar et al. (2011) [17].
(TIF)

Figure S2 **Sequence analysis of HaRxL44.** (A) Alignment of HaRxL44 from *Hpa* with predicted effector from *P. infestans* PITG_04266 (EEY67272) and PITG_07586 (EEY53937) and from *P. sojae* (Avh109). (B) Schematic representation of the *Hpa* genomic region where *HaRxL44* gene is found. Retro-transposons are represented in black, while grey boxes represent non-RxLR encoding genes. *HaRxL* effector candidate genes are represented in colour. Scale shows the number of base pair. (C) Nonsynonymous amino acid polymorphisms in HaRxL44 among the *Hpa* isolates. Predicted signal peptide (SP) at the N-terminus, followed by a host-targeting sequence (HTS) and bipartite-nucleolar localisation signal (NoLs) are indicated.
(TIF)

Figure S3 ***Arabidopsis*** **transgenic lines expressing** **Δ*SP-HaRxL44*** **under the control of different plant promoters.** (A) RT-PCR on *HaRxL44* transcript in transgenic lines expressing HaRxL44 under the control of 35S promoter (44 lines), compared with WT. *EF1a* is used as loading control. (B) GUS staining in *Hpa*-infected leaf in plant expressing GUS under the control of dP2, a “haustoriated-cell specific” promoter (proMAP65-3) (Quentin et al., unpublished data). (C) Western blot (anti-GFP) on proteins extracted from transgenic lines expressing GFP-HaRxL44 or GFP under the control of dP2 promoter. Note the enrichment of GFP-HaRxL44 6 DAI *Hpa* compared to mock-treated plant. (D) Western blot (anti-GFP) on proteins extracted from two independent transgenic lines expressing *HaRxL44-GFP* under the control of DEX inducible promoter (D44-lines) upon DEX treatment.
(TIF)

Figure S4 **Western blot analysis on proteins extracted from transgenic lines expressing or not GFP-MED19a, using GFP antibody.**
(TIF)

Figure S5 (A) Co-immunoprecipitation assay using GFP beads on protein extracted after transient expression in *N. benthamiana* of GFP-MED19a and RFP-tagged nuclear-HaRxLs (or RFP). Note that in the presence of RFP-HaRxL44, GFP-MED19a signal is reduced in both input and IP (arrows). (B) RT-PCR on *MED19a* transcript in two transgenic lines expressing HaRxL44 compared to Col-0. *EF1a* is used as loading control. (C) Monitoring of *Hpa* Waco9 sporulation at 5 day after inoculation in mutant lines *boi* RNAi and *mbr1-like* KO line. Error bars represent the standard error of the mean. Asterisks represent the significance of individual unpaired *t* tests comparing the given column with the control. (D) Western blot on proteins extracted from four independent Arabidopsis transgenic lines expressing both GFP-MED19a and HA-HaRxL44 (or HA-GUS) ± MG132. (E) Immunoblotting of protein extracted from *Arabidopsis* leaves after Co-IP assay. Note the Co-IP of MED6 with GFP-MED19a even in the presence of HA-HaRxL44.
(TIF)

Figure S6 **Mutagenesis of HaRxL44 allowed the identification of a mutant allele.** (A) Sequence of HaRxL44 from SP cleavage site. RxLR motif and predicted bipartite-nucleolar localisation signal are indicated. The mutagenized amino acids are highlighted in red, and the corresponding mutant is underlined in black. (B) Localization of the HaRxL44 mutant alleles determined by confocal microscopy. (C) Western blot analysis on proteins extracted from *N. benthamiana* leave expressing HaRxL44 mutant alleles. (D) Sequence of the HaRxL44^M^ used in Figure 4, corresponding to HaRxL44 M4 in Figure S6A–C.
(TIF)

Figure S7 **HaRxL44 expression** ***in planta*** **induced JA/ET-dependent defence.** (A–B) qRT-PCR on *JAZ1 and JAR1* marker genes in 5-wk-old 44-lines compared to WT. Data are presented as average fold induction compared with control of three biological replicates ± SD. (C) Mapman representation of the JA biosynthesis pathway. Note the induction of two genes, *OPR3* (AT2G06050) and *LOX2* (AT3G45140), in transgenic lines expressing HaRxL44 under the control of 35S promoter (44 lines). (D) Representative picture of *B. cinerea* symptoms 5 DAI in 44 lines compared to Col-0. (E) Representative picture of *B. cinerea* symptoms 5 DAI in *Arabidopsis* transgenic lines expressing HaRxL44 under the control of DEX inducible promoter (D44 #1 and D44 #2) in the presence or not of dexamethazone and in lines expressing HaRxL44 under the 35S promoter (44 #1 and 44 #2). (F) Representative picture of *B. cinerea* symptoms under UV light 5 DAI in *Arabidopsis* transgenic lines expressing D44 in the presence of increasing amount of DEX. Note that the increase amounts of GFP signal (yellow) corresponding to GFP-HaRxL44 expression upon DEX treatment is correlated with the reduction of *B. cinerea* lesion size.
(TIF)

Figure S8 **HaRxL44 expression and** ***Hpa*** **suppress** ***PR1*** **expression.** (A–B) qRT-PCR on SA marker genes (*PR2* and *PR5*) in 5-wk-old transgenic lines expressing HaRxL44 under the control of 35S promoter (44 lines) in comparison to Col-0. Data are presented as average fold induction compared with control of three biological replicates ± SD. (C) GUS staining of pro(*PR1*)::GUS in *Arabidopsis* leaves, 3 DAI *Hpa* Waco9. (D) Co-staining of GUS (dark blue) and *Hpa* hyphae (green) using Anilin blue staining in *PR1*::GUS line 3 DAI *Hpa*. Red arrows indicate SA induction in vascular tissues.
(TIF)

Table S1 **Correlation between MeJA-responsive genes and differentially expressed genes in HaRxL44-lines and 3 DAI with Hpa Waco9.**
(XLSX)

Table S2 **Expression of MeJA-regulated gene in HaRxL44-lines and 3 DAI with** ***Hpa*** **Waco9.**
(XLSX)

## Abbreviations

BiFC: Bimolecular Fluorescence Complementation
BOI: BOTRYTIS SUSCEPTIBLE 1
BOS1: *BOTRYTIS SUSCEPTIBLE 1*
Co-IP: co-immunoprecipitation
COR: coronatine
DAI: day after infection
DEX: dexamethazone
ET: ethylene
ETI: effector-triggered immunity
HaRxLs: *Hpa* RXLR effectors
*Hpa*: *Hyaloperonospora arabidopsidis*
HTS: host-targeting sequence
HUB1: HISTONE MONOUBIQUITINATION1
JA: jasmonic acid
KO: knock out
MBR1-like: MED25-BINDING RING-H2 PROTEIN-like
MED19a: Mediator subunit 19a
MeJA: methyl jasmonic acid
NoLs: nucleolar localisation signal
*NPR1*: NON-EXPRESSOR OF PATHOGENESIS-RELATED GENES1
*Pst*: *Pseudomonas syringae* pv. tomato
PTI: pattern-triggered immunity
SA: salicylic acid
SAR: systemic acquired resistance
SATI: SA-triggered immunity
T3S: type three secretion
WT: wild-type
Y2H: yeast 2-hybrid
