# Correction: A Downy Mildew Effector Attenuates Salicylic Acid–Triggered Immunity in Arabidopsis by Interacting with the Host Mediator Complex

**DOI:** 10.1371/journal.pbio.1001909

**Published:** 2014-06-25

**Authors:** The PLOS Biology Staff

Since publication of this paper, we became aware of several incorrect or missing details that required correcting. These are as follows:

## 1. Figure 5 correction 1 – changed panel

In the published paper, the HA input blot in figure 4C was wrongly duplicated in figure 5A. This error came from the fact that figure 4C and 5A display results obtained from the same experiment, but the samples were loaded in a different order, which resulted in mislabelling in figure 5A. In figure 5A, the duplicated HA input blot has now been replaced with the appropriate one.

**Figure 5:**
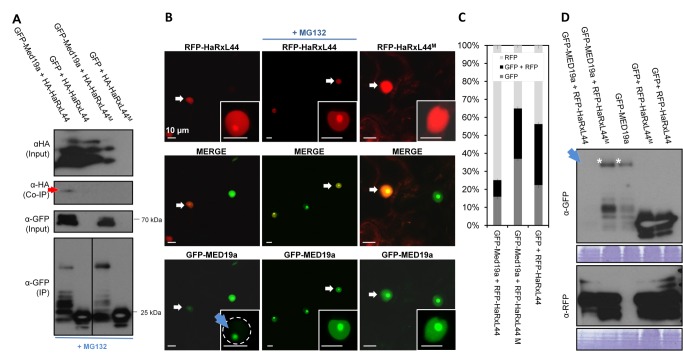
Interaction between MED19a and HaRxL44 is important for HaRxL44–induced MED19a degradation via proteasome. (A) Immunoblotting of proteins, extracted from *N. benthamiana* leaves after transient assay. Note the absence of Co-IP of HA-HaRxL44^M^ with GFP-MED19a. A dividing line was added to the original IP blot for the bottom panel to indicate which lanes were spliced together. (B) Co-localisation analysis between GFP-MED19a and RFP-HaRxL44 or HaRxL44^M^ determined by transient assay in *N. benthamiana*. Note the lack of GFP-MED19a in the presence of RFP-HaRxL44 (arrow) but not HaRxL44^M^. (C) Quantification of the number of fluorescent nucleoplasm observed in nucleus transformed with GFP-MED19a in the presence or not of RFP-HaRxL44 or RFP-HaRxL44^M^. All the confocal pictures were taken with PMT 1 (494–541 nm) at Gain: 864 and PMT 2 (591–649 nm) at Gain: 844. Note the decrease in GFP-MED19a transformed cells in the presence of RFP-HaRxL44 in comparison with RFP alone or RFP-HaRxL44^M^. (D) Immunoblotting of protein extracted from *N. benthamiana* leaves after transient expression of GFP-MED19a alone or with RFP-HaRxL44, RFP-HaRxL44M. GFP co-infiltrated with RFP-HaRxL44 or RFP-HaRxL44M was used as control.

## 2. Figure 5 correction 2 – corrected labelling and explanation regarding splice line added

In figure 5A, the third blot down that was previously incorrectly labelled ‘IP’ is now labelled ‘input GFP’. In this blot, only GFP MED19a is visible; no GFP alone is shown because the blot is overexposed at the lower molecular weight. Because the samples were loaded in the wrong order, the fourth (lowest) panel, labelled ‘IP GFP’ in figure 5A, shows lanes that were spliced to position them to generate the figure showing the lanes in the same relative order as the other blots. This information has been included in the revised legend of figure 5A.

## 3. Figure 5 correction 3 – missing information regarding panel D added

In the version of the article initially published, the following sentence was missing from the legend to figure 5: "(D) Immunoblotting of protein extracted from N. benthamiana leaves after transient expression of GFP MED19a alone or with RFP-HaRxL44, or RFP-HaRxL44M. GFP co-infiltrated with RFP-HaRxL44 or RFP-HaRxL44M was used as control."

## 4. Materials and Methods correction - information corrected

We also noticed that in the Methods section “Protein Extraction, Co-Immunoprecipitation, and Western Blot”, the extraction buffer was initially incorrectly described; the concentration of some components corresponded to the concentration of the stock solution used to prepare the buffer and not the final concentrations.

For reasons of accuracy, the sentence –

“extraction buffer (4-10 mL) [1 M Tris HCl pH7.5, 5 M NaCl, 0.5M EDTA, 20% glycerol, 10 mM DTT, 1 X Protease inhibitor (Sigma), 20% Triton X-100, 2 % PVPP]”

should read:

"extraction buffer [20 mM Tris-HCl pH 7.5, 150 mM NaCl, 1 mM EDTA, 10% glycerol, 10 mM DTT, 1X Protease inhibitor (Sigma), 2% Triton X-100, 2% PVPP]".

NB: As an additional note, reference number 100 was under revision at the time of publication. This paper has now been published and is available here:

Rallapalli G, Kemen EM, MacLean D, Etherington G, Jones JDG (2014) Expression profiling through randomly sheared cDNA tag sequencing (EXPRSS) reveals transcriptional dynamics in plant defense networks. http://www.biomedcentral.com/1471-2164/15/341
