# Correction: A Downy Mildew Effector Attenuates Salicylic Acid-Triggered Immunity in Arabidopsis by Interacting with the Host Mediator Complex

**DOI:** 10.1371/journal.pbio.1002408

**Published:** 2016-03-02

**Authors:** Marie-Cécile Caillaud, Shuta Asai, Ghanasyam Rallapalli, Sophie Piquerez, Georgina Fabro, Jonathan D. G. Jones

We, the authors, would like to correct aspects of the manuscript text and Fig 2, in order to clarify some issues that have been raised by readers.

**Fig 2:**
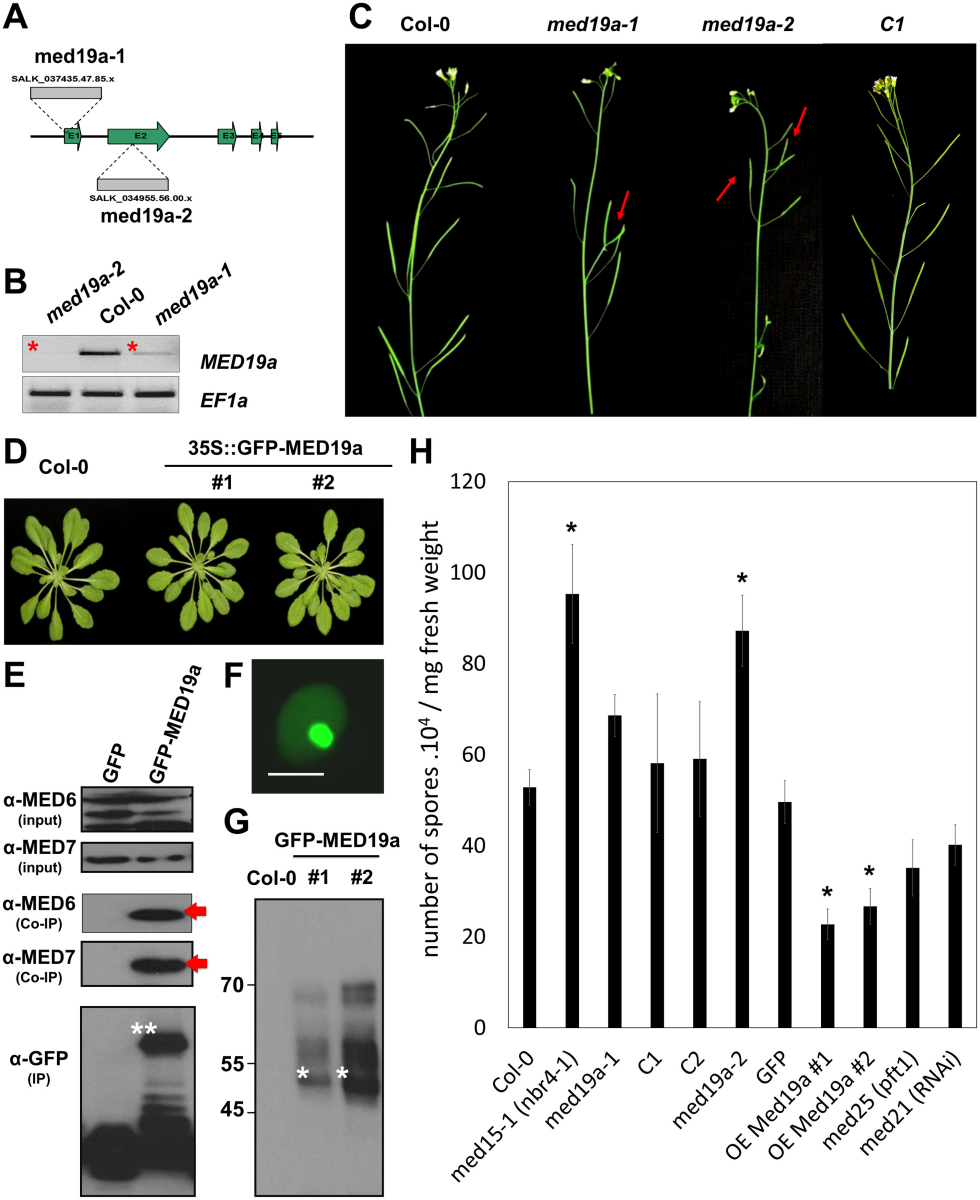
MED19a is a positive regulator of nuclear immunity against *Hpa*. (A) Schematic diagram of T-DNA insertions in *MED19a*. (B) *MED19a* expression in *med19a-1* and *med19a-2* mutants. (C) Representative images of the phenotype observed in 4-wk-old floral stem of Col-0, *med19a-1*, *med19a-2*, and *med19a* mutant complemented line C1. (D) Developmental phenotype of *Arabidopsis* transgenic lines OE-MED19a compared to Col-0. (E) Immunoblot of the Co-immunoprecipitation analysis between GFP-MED19a and MED6 and MED7. Arrows point out the interaction detected between GFP-MED19a and MED6 and MED7. (F) Subcellular localisation of GFP-MED19a in *Arabidopsis* plant. Scale bar, 5 μm. (G) Immunoblot of proteins extracted from two independent lines expressing GFP-MED19a. Stars indicate the expected size for GFP-MED19a. Notice the upper bands in the blot that might suggest posttranscriptional modifications. (H) Monitoring of *Hpa* sporulation at 5 DAI in control lines (Col-0 and GFP), *med19a* mutant complemented lines (C1 and C2), Mediator mutants, and MED19a OE lines. Error bars represent the standard error of the mean. Asterisks represent the significance at P < 0.01 (pairwise t-test, with Holm correction, after mixed effect linear model; ANOVA with Tukey’s HSD).

1 After reanalysis, we accept that the small differences in Fig 2H between Col-0 and *med19a-1* are not significant. We accept we therefore now cannot claim significance for differences between Col-0 and *med19a-1* but the claimed significance for *med19a-2*, OE-Med19a #1 and OE-Med19a #2 remain valid. The weaker phenotype observed for *med19a-1* mutant in response to *Hpa* (compared with med19a-2) is consistent with the RT-PCR displayed in fig 2B, where transcript for *Med19a* could still be observed in the *med19a-1* mutant and with the discussion of the paper where we also point out that it should be borne in mind that the phenotype observed for med19a KO mutants may be only partial, because MED19a and MED19b could have redundant functions.

We therefore would like to correct the following in the manuscript:

In the Results section subheading *HaRxL44 targets MED19a*, *a Positive Regulator of Plant Immunity to Hpa*, the second sentence of the fifth paragraph should read: The *med19a-2* mutant was more susceptible to *Hpa* than wild type, similar to a *med15* mutant, which has impaired SATI (*med15*[37]; Fig 2H).

2 We’d like to correct Fig 2 in order to remove the asterisk from the graph in panel H showing the sporulation of *Hpa* on med19a-1, and to correct the legend for Fig 2. The corrected legend and figure are provided here.

The reanalysis of the data also showed that the differences in Fig 2H between Col-0 and *med21* and *med25* KO mutants are not significant. We used these well described regulators of plant defence as control for the experiment. We acknowledge that the number of observations is not sufficient to claim that there is a difference between Col-0 and *med21* and *med25* KO. However, this result does not impact the overall message of the paper which shows that HaRxL44 interferes with Mediator function by degrading MED19, shifting the balance of defence transcription from SA-responsive defence to JA/ET-signalling, and enhancing susceptibility to biotrophs by attenuating SA-dependent gene expression.

We therefore correct our interpretation of the Hpa susceptibility of *med21* and *med25* KO mutants in the manuscript, as follows:

In the Results section, subheading *JA/ET Signalling Is Induced in the Presence of HaRxL44*, *the Absence of MED19a*, *and 3 d After Hpa Infection*, the penultimate sentence of the final paragraph should read: We did not observe in either *med21* RNAi line and *med25* knock out (KO) mutants that *Hpa* growth was reduced compared with WT (Fig 2H)”. The final sentence of the paragraph should be removed.

3 We would also like to add the following sentence to subheading *Protein Extraction*, *Co-Immunoprecipitation*, *and Western Blot* of the Methods section: Blots were stained with Coomassie (Instant Blue, Expedeon) to visualize protein loading in the figure.
4 We would like to correct an error in the legend for Fig 3 to show the correct panels: A, B & C. The corrected legend is provided here.
5 Finally, we would like to add the follow sentence to the Acknowledgements section: We acknowledge Dan MacLean (TSL, Norwich) for help improving the quality of the statistical analysis.

**Fig 3:**
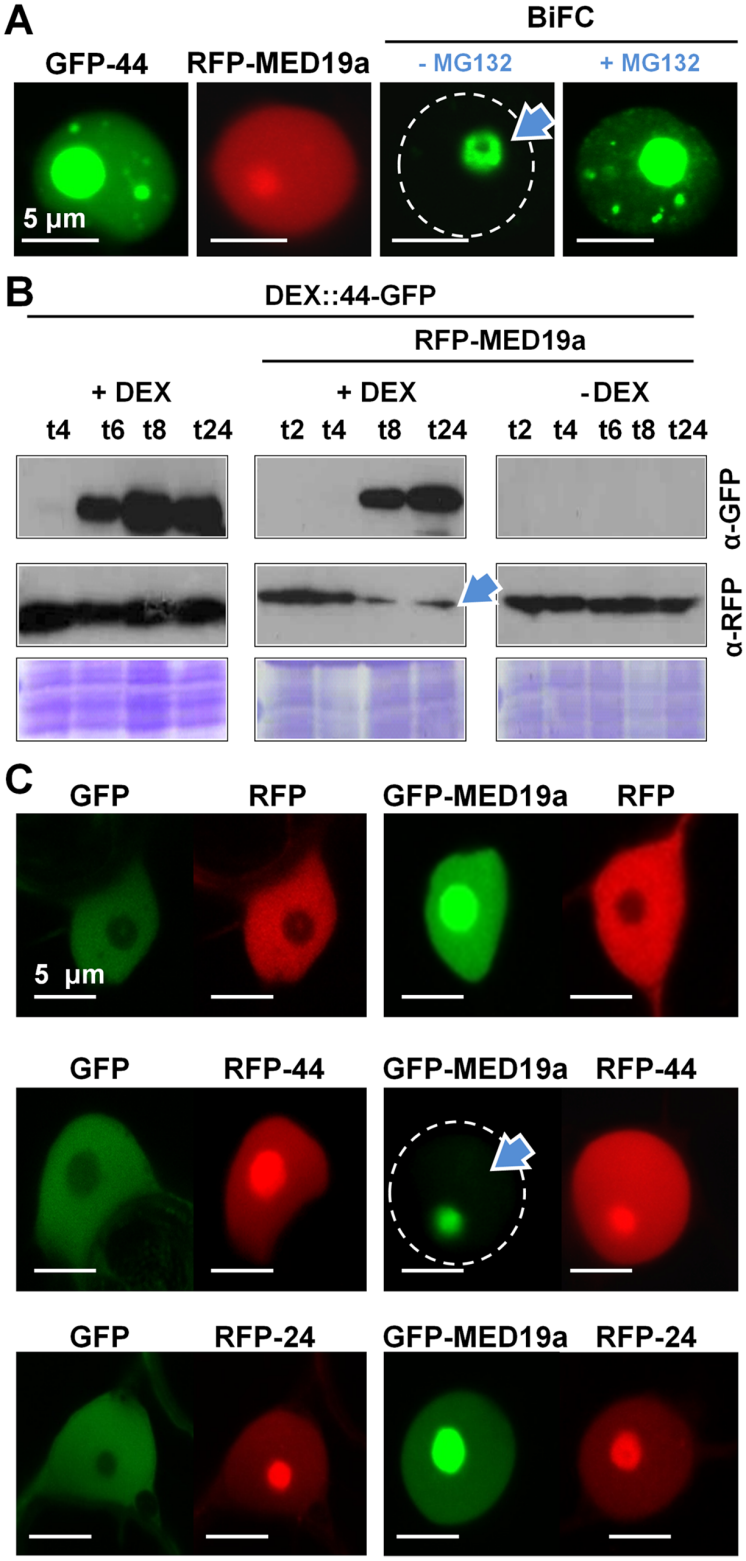
HaRxL44 destabilizes MED19a *in planta*. (A) Subcellular localisation of GFP-HaRxL44 (in green), RFP-MED19a (in red), and YFPc-HaRxL44 + YFPn-MED19a (BiFC, yellow) obtained by transient expression in *N*. *benthamiana*. n, nucleus. (B) Western blot analysis of protein extracted after transient expression of DEX::HaRxL44-GFP with RFP or RFP-MED19a in the presence or not of dexamethasone (DEX). Note the decrease in the level of MED19a observed in the presence of HaRxL44. (C) Co-localisation analysis between GFP-MED19a and nuclear-HaRxLs determined by transient assay in *N*. *benthamiana*. Note the lack of GFP-MED19a in the presence of RFP-HaRxL44 (arrow).
