# Supplementary figures and images for: A Downy Mildew Effector Attenuates Salicylic Acid–Triggered Immunity in Arabidopsis by Interacting with the Host Mediator Complex
Source: PLoS Biol. 2013 Dec 10;11(12):e1001732. doi: 10.1371/journal.pbio.1001732 (PMC3858237; doi:10.1371/journal.pbio.1001732)

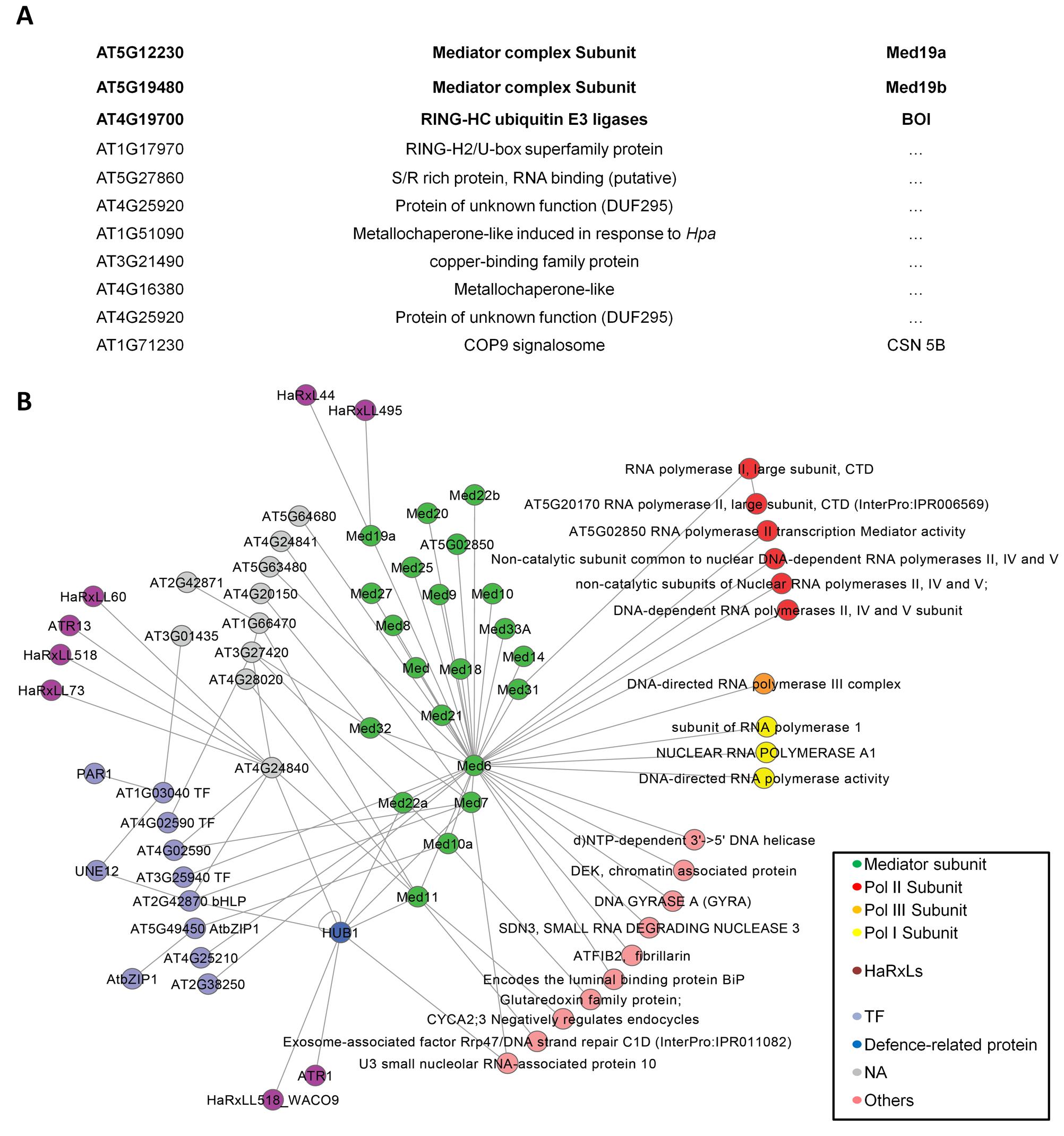

Supplement: Figure S1 — Interactomic data extracted from Mukhtar et al. (2011) [17] . (A) List of the plant proteins interacting with HaRxL44 in Y2H. (B) Cytoscape representation of the network of the interactions obtained in Y2H for Mediator subunits. Data extracted from Mukhtar et al. (2011) [17]. (TIF) [file pbio.1001732.s001.tif]

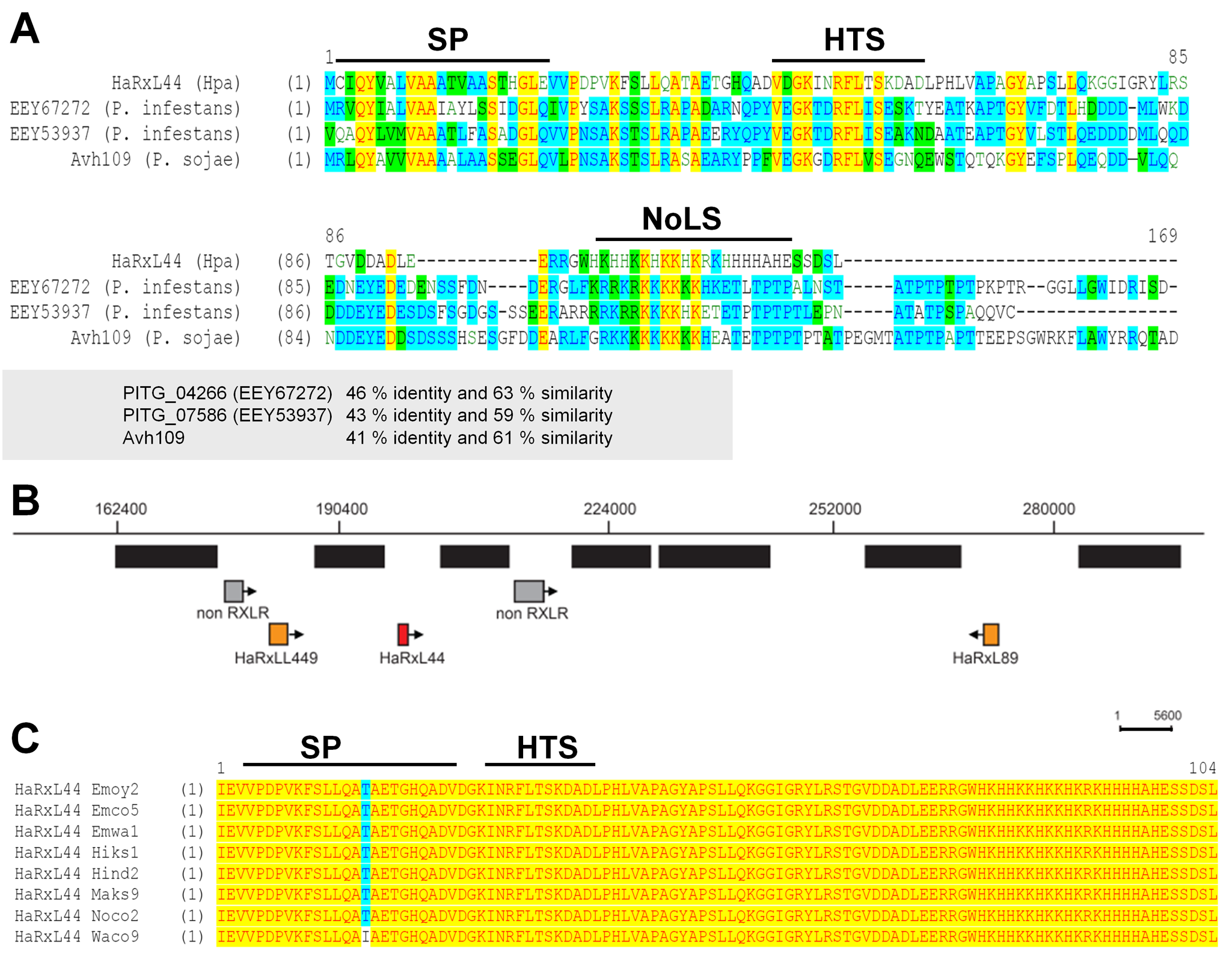

Supplement: Figure S2 — Sequence analysis of HaRxL44. (A) Alignment of HaRxL44 from Hpa with predicted effector from P. infestans PITG_04266 (EEY67272) and PITG_07586 (EEY53937) and from P. sojae (Avh109). (B) Schematic representation of the Hpa genomic region where HaRxL44 gene is found. Retro-transposons are represented in black, while grey boxes represent non-RxLR encoding genes. HaRxL effector candidate genes are represented in colour. Scale shows the number of base pair. (C) Nonsynonymous amino acid polymorphisms in HaRxL44 among the Hpa isolates. Predicted signal peptide (SP) at the N-terminus, followed by a host-targeting sequence (HTS) and bipartite-nucleolar localisation signal (NoLs) are indicated. (TIF) [file pbio.1001732.s002.tif]

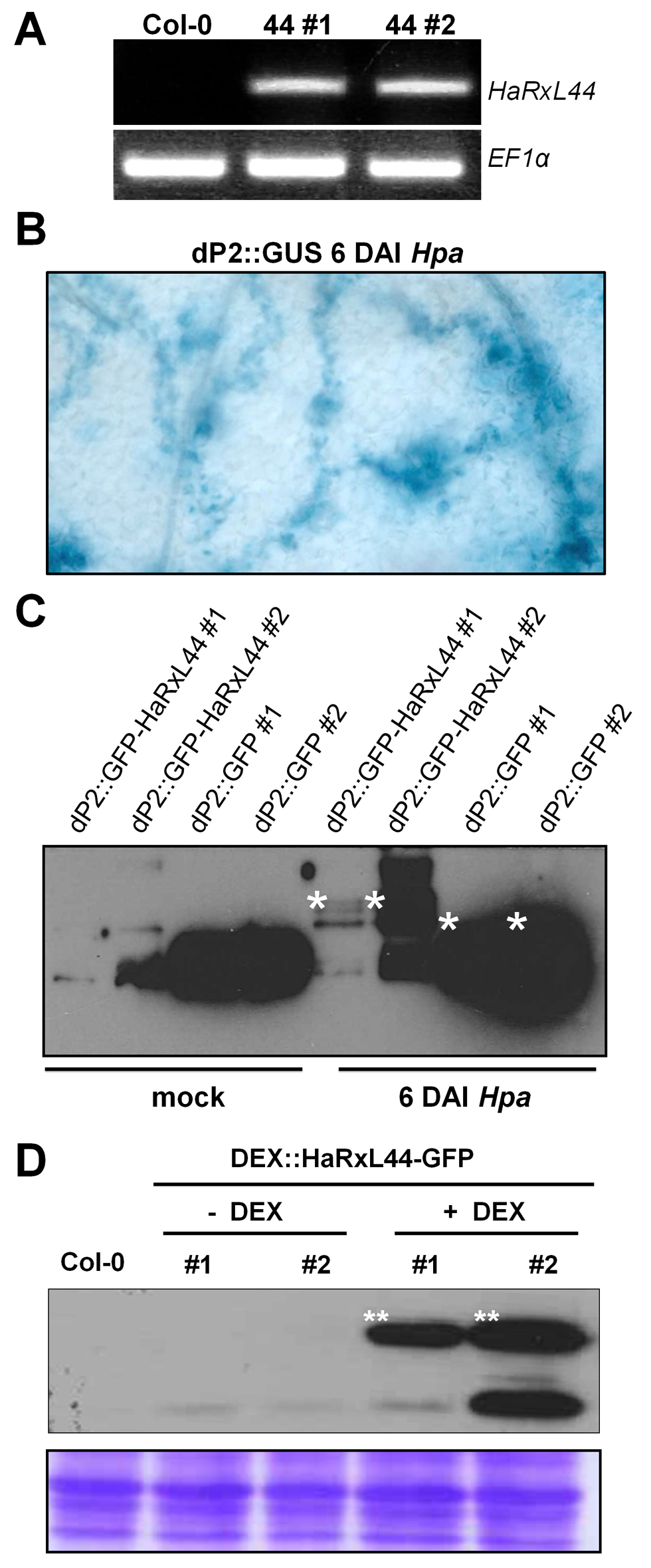

Supplement: Figure S3 — Arabidopsis transgenic lines expressing ΔSP-HaRxL44 under the control of different plant promoters. (A) RT-PCR on HaRxL44 transcript in transgenic lines expressing HaRxL44 under the control of 35S promoter (44 lines), compared with WT. EF1a is used as loading control. (B) GUS staining in Hpa-infected leaf in plant expressing GUS under the control of dP2, a “haustoriated-cell specific” promoter (proMAP65-3) (Quentin et al., unpublished data). (C) Western blot (anti-GFP) on proteins extracted from transgenic lines expressing GFP-HaRxL44 or GFP under the control of dP2 promoter. Note the enrichment of GFP-HaRxL44 6 DAI Hpa compared to mock-treated plant. (D) Western blot (anti-GFP) on proteins extracted from two independent transgenic lines expressing HaRxL44-GFP under the control of DEX inducible promoter (D44-lines) upon DEX treatment. (TIF) [file pbio.1001732.s003.tif]

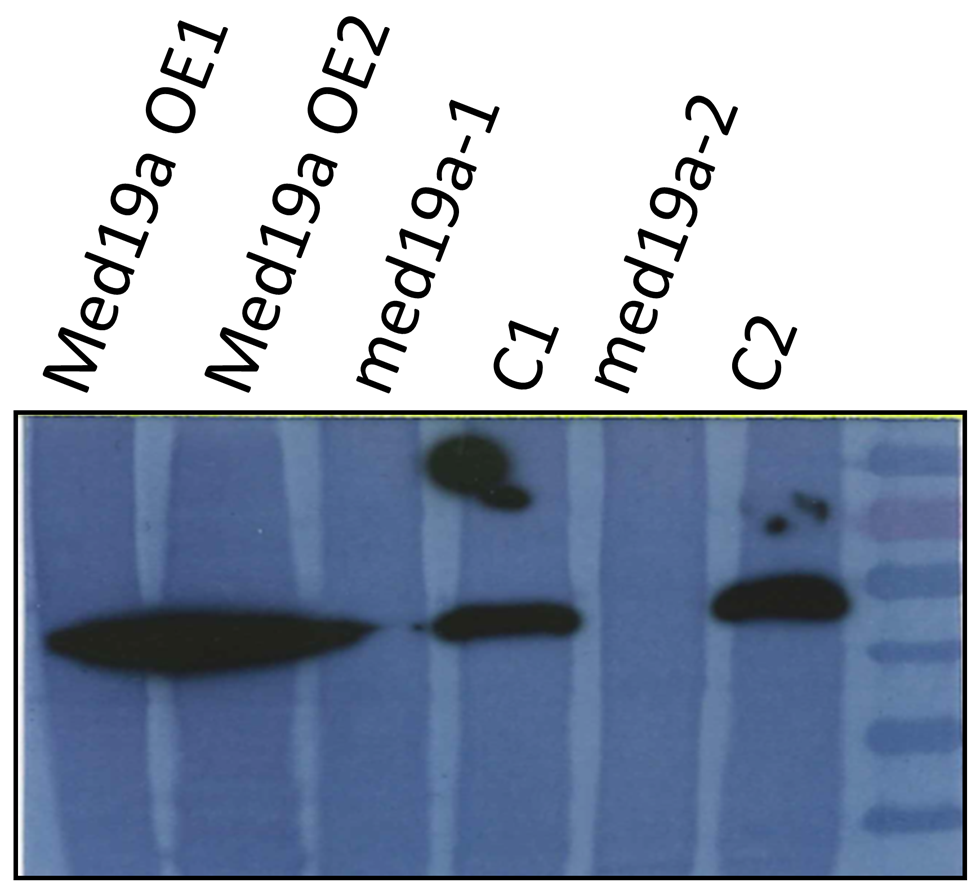

Supplement: Figure S4 — Western blot analysis on proteins extracted from transgenic lines expressing or not GFP-MED19a, using GFP antibody. (TIF) [file pbio.1001732.s004.tif]

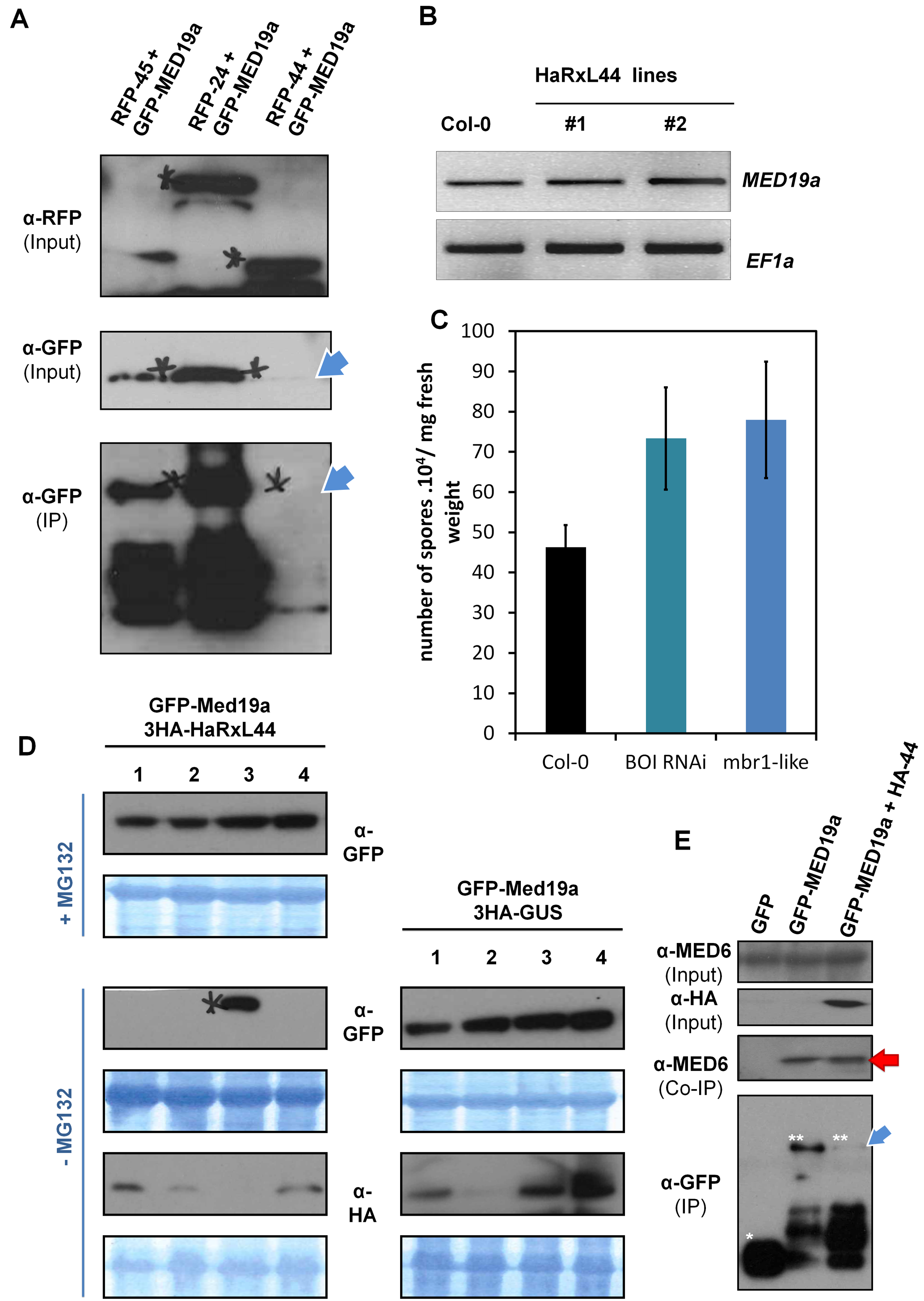

Supplement: Figure S5 — (A) Co-immunoprecipitation assay using GFP beads on protein extracted after transient expression in N. benthamiana of GFP-MED19a and RFP-tagged nuclear-HaRxLs (or RFP). Note that in the presence of RFP-HaRxL44, GFP-MED19a signal is reduced in both input and IP (arrows). (B) RT-PCR on MED19a transcript in two transgenic lines expressing HaRxL44 compared to Col-0. EF1a is used as loading control. (C) Monitoring of Hpa Waco9 sporulation at 5 day after inoculation in mutant lines boi RNAi and mbr1-like KO line. Error bars represent the standard error of the mean. Asterisks represent the significance of individual unpaired t tests comparing the given column with the control. (D) Western blot on proteins extracted from four independent Arabidopsis transgenic lines expressing both GFP-MED19a and HA-HaRxL44 (or HA-GUS) ± MG132. (E) Immunoblotting of protein extracted from Arabidopsis leaves after Co-IP assay. Note the Co-IP of MED6 with GFP-MED19a even in the presence of HA-HaRxL44. (TIF) [file pbio.1001732.s005.tif]

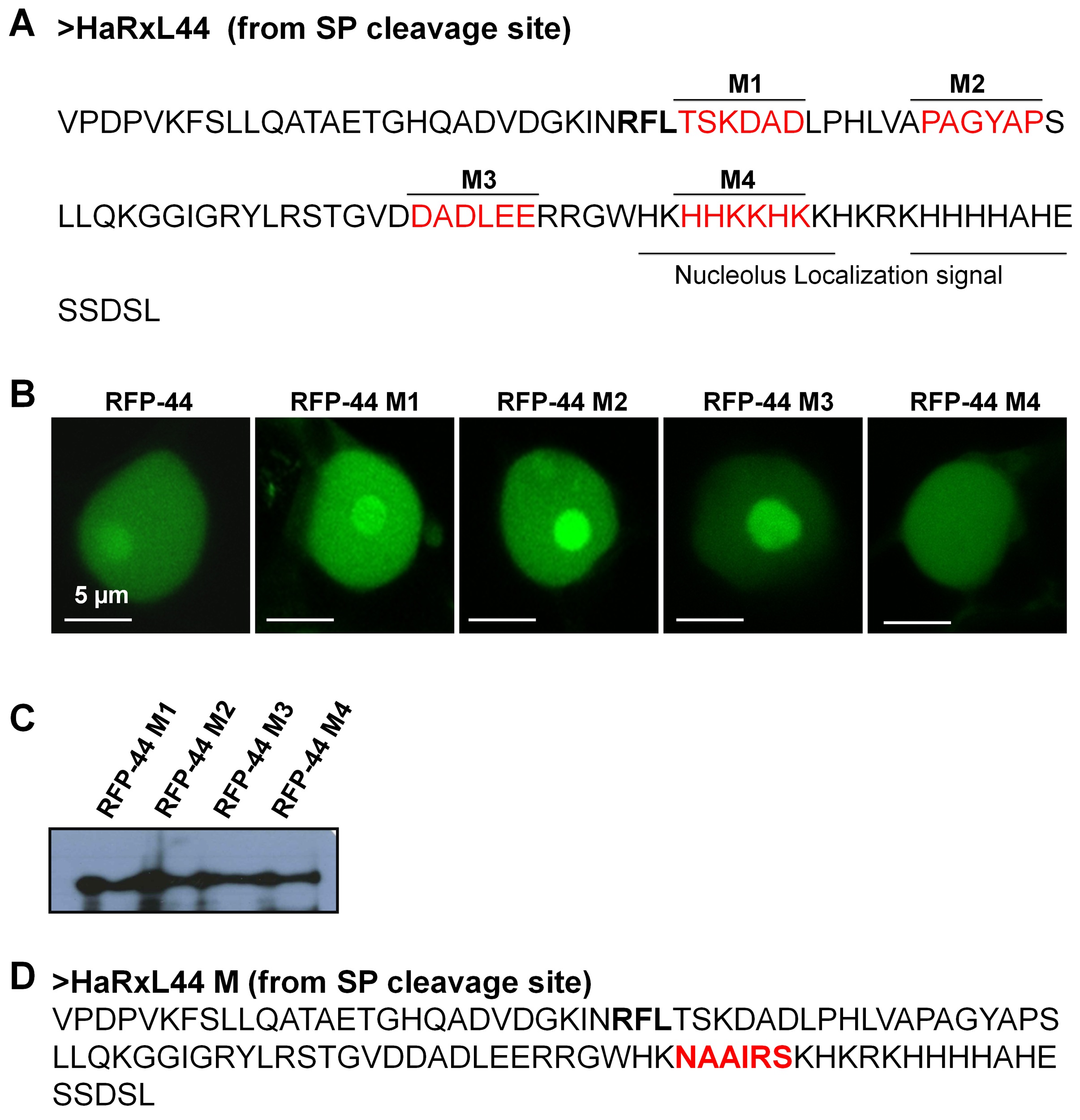

Supplement: Figure S6 — Mutagenesis of HaRxL44 allowed the identification of a mutant allele. (A) Sequence of HaRxL44 from SP cleavage site. RxLR motif and predicted bipartite-nucleolar localisation signal are indicated. The mutagenized amino acids are highlighted in red, and the corresponding mutant is underlined in black. (B) Localization of the HaRxL44 mutant alleles determined by confocal microscopy. (C) Western blot analysis on proteins extracted from N. benthamiana leave expressing HaRxL44 mutant alleles. (D) Sequence of the HaRxL44M used in Figure 4, corresponding to HaRxL44 M4 in Figure S6A–C. (TIF) [file pbio.1001732.s006.tif]

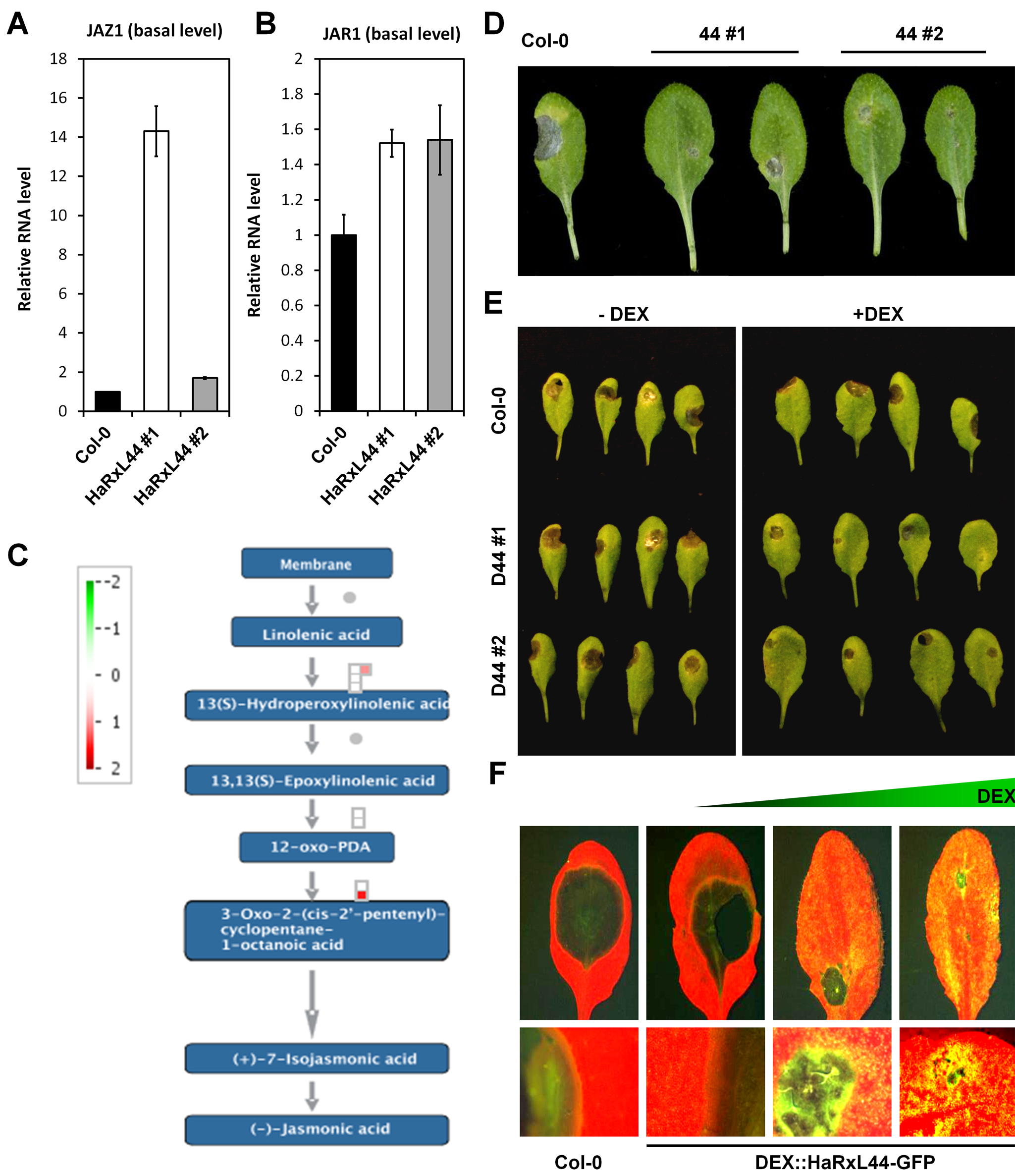

Supplement: Figure S7 — HaRxL44 expression in planta induced JA/ET-dependent defence. (A–B) qRT-PCR on JAZ1 and JAR1 marker genes in 5-wk-old 44-lines compared to WT. Data are presented as average fold induction compared with control of three biological replicates ± SD. (C) Mapman representation of the JA biosynthesis pathway. Note the induction of two genes, OPR3 (AT2G06050) and LOX2 (AT3G45140), in transgenic lines expressing HaRxL44 under the control of 35S promoter (44 lines). (D) Representative picture of B. cinerea symptoms 5 DAI in 44 lines compared to Col-0. (E) Representative picture of B. cinerea symptoms 5 DAI in Arabidopsis transgenic lines expressing HaRxL44 under the control of DEX inducible promoter (D44 #1 and D44 #2) in the presence or not of dexamethazone and in lines expressing HaRxL44 under the 35S promoter (44 #1 and 44 #2). (F) Representative picture of B. cinerea symptoms under UV light 5 DAI in Arabidopsis transgenic lines expressing D44 in the presence of increasing amount of DEX. Note that the increase amounts of GFP signal (yellow) corresponding to GFP-HaRxL44 expression upon DEX treatment is correlated with the reduction of B. cinerea lesion size. (TIF) [file pbio.1001732.s007.tif]

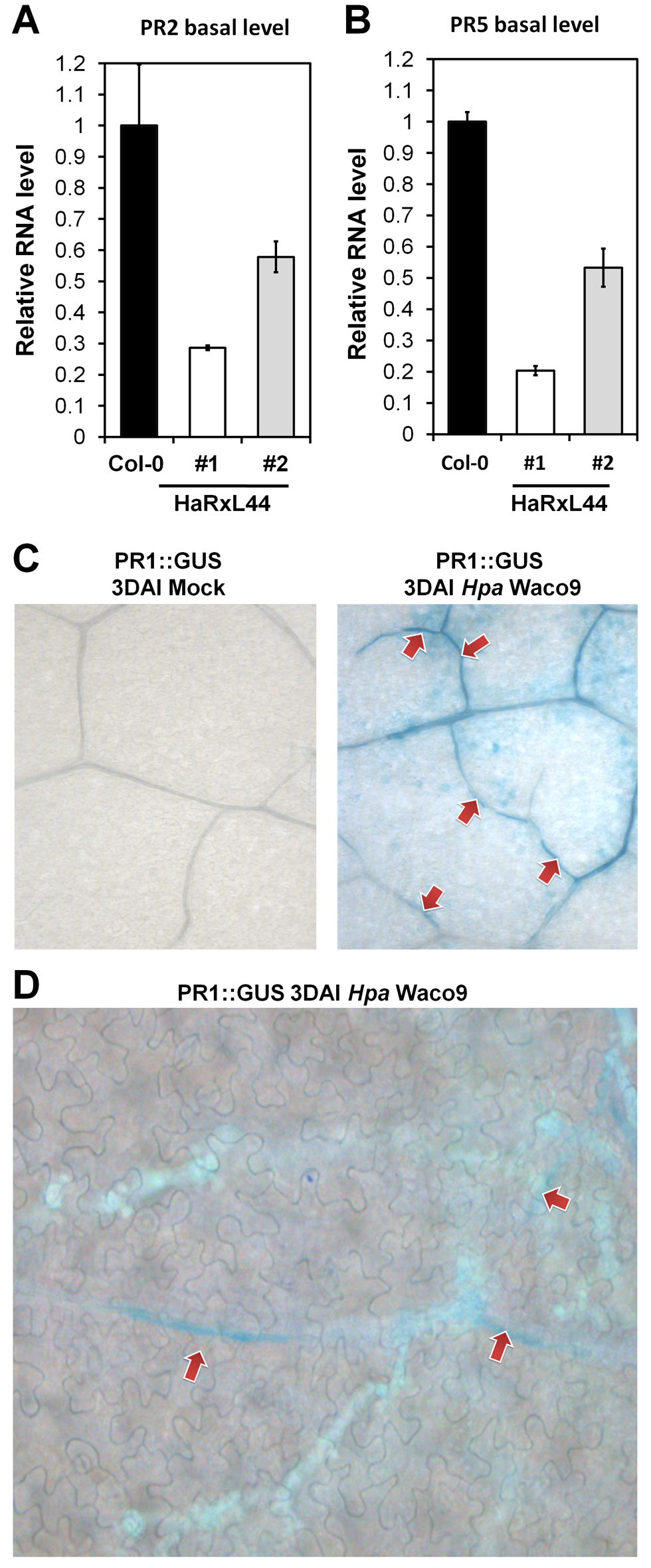

Supplement: Figure S8 — HaRxL44 expression and Hpa suppress PR1 expression. (A–B) qRT-PCR on SA marker genes (PR2 and PR5) in 5-wk-old transgenic lines expressing HaRxL44 under the control of 35S promoter (44 lines) in comparison to Col-0. Data are presented as average fold induction compared with control of three biological replicates ± SD. (C) GUS staining of pro(PR1)::GUS in Arabidopsis leaves, 3 DAI Hpa Waco9. (D) Co-staining of GUS (dark blue) and Hpa hyphae (green) using Anilin blue staining in PR1::GUS line 3 DAI Hpa. Red arrows indicate SA induction in vascular tissues. (TIF) [file pbio.1001732.s008.tif]
